# Direct pharmacological targeting of RAC1 by chlorogenic acid: A novel therapeutic approach for mesangial proliferative glomerulonephritis

**DOI:** 10.1016/j.apsb.2026.06.041

**Published:** 2026-06-24

**Authors:** Yilun Qu, Lingling Wu, Yong Wang, Fei Peng, Jie Zhang, Dan Pan, Tiantian Wang, Yena Zhou, Jikai Xia, Jiayi He, Chunru Shi, Yinghua Zhao, Xu Wang, Ran Liu, Yingjie Zhang, Jiaona Liu, Jie Wu, Shuwei Duan, Guangyan Cai, Ping Li, Quan Hong, Xiangmei Chen

**Affiliations:** a Senior Department of Nephrology, Chinese PLA General Hospital, State Key Laboratory of Kidney Diseases, National Clinical Research Center for Kidney Diseases, Beijing Key Laboratory of Medical Devices and Integrated Traditional Chinese and Western Drug Development for Severe Kidney Diseases, Beijing Key Laboratory of Digital Intelligent TCM for the Prevention and Treatment of Pan-vascular Diseases, Key Disciplines of National Administration of Traditional Chinese Medicine, Innovation Team and Talents Cultivation Program of National Administration of Traditional Chinese Medicine, Beijing 100853, China; b School of Medicine, Nankai University, Tianjin 300071, China; c Institute of Chinese Medicine, Guangdong Pharmaceutical University, Guangzhou 510006, China; d Clinical Research Center, The First Affiliated Hospital of Shantou University Medical College, Shantou 515041, China; e Beijing Tsinghua Changgung Hospital, School of Clinical Medicine, Tsinghua University, Beijing 102218, China

**Keywords:** Chlorogenic acid, Inflammatory response, Mesangial cell activation, Mesangial proliferative glomerulonephritis, RAC1, AKT, Thrombospondin-1, CD36

## Abstract

Mesangial proliferative glomerulonephritis (MsPGN) is a common cause of end-stage renal disease, characterized by mesangial cell proliferation within glomeruli. Mesangial cell activation triggered by inflammation is a key factor in the development of MsPGN. However, effective therapeutic strategies targeting this process are still limited. Here, we uncovered, for the first time, the direct effects of chlorogenic acid (CGA), a naturally occurring small-molecule compound with anti-inflammatory and antiproliferative properties, on mesangial cells in an anti-Thy1 nephritis animal model. A multi-dimensional pharmacological platform integrating laser microdissection-coupled glomerular proteomics affinity deconvolution, surface plasmon resonance, molecular dynamics, and enzyme assays identified Ras-related C3 botulinum toxin substrate 1 (RAC1) as the direct target of CGA. Mechanistically, CGA competitively binds to the LYS15, PRO33, and THR34 amino acid residues—located within residues 57–65 of the GTP/GDP-binding domain of RAC1, inhibiting its activation and subsequently reducing AKT phosphorylation while suppressing Thrombospondin-1 secretion from mesangial cells—a key ligand for macrophage CD36 receptors. This interaction deactivates macrophages and lowers the levels of inflammatory cytokines, including TNF*α*, IL1*β*, and IL6. Significantly, as a novel natural RAC1 inhibitor, CGA disrupts mesangial-macrophage crosstalk by dual suppression of regional immunity and cellular proliferation, thus conferring renal protection in MsPGN models. Our findings highlight CGA as a promising pharmacotherapy, offering a mechanism-driven, natural product-based strategy to mitigate MsPGN progression.

## Graphical abstract

CGA treatment attenuates MsPGN. As a natural RAC1 inhibitor, CGA confers dual anti-inflammatory and anti-proliferative protection in glomeruli by targeting the RAC1–AKT–THBS1–CD366 signaling axis, thereby providing novel mechanistic insights for therapeutic strategies against MsPGN.

**Figure:**
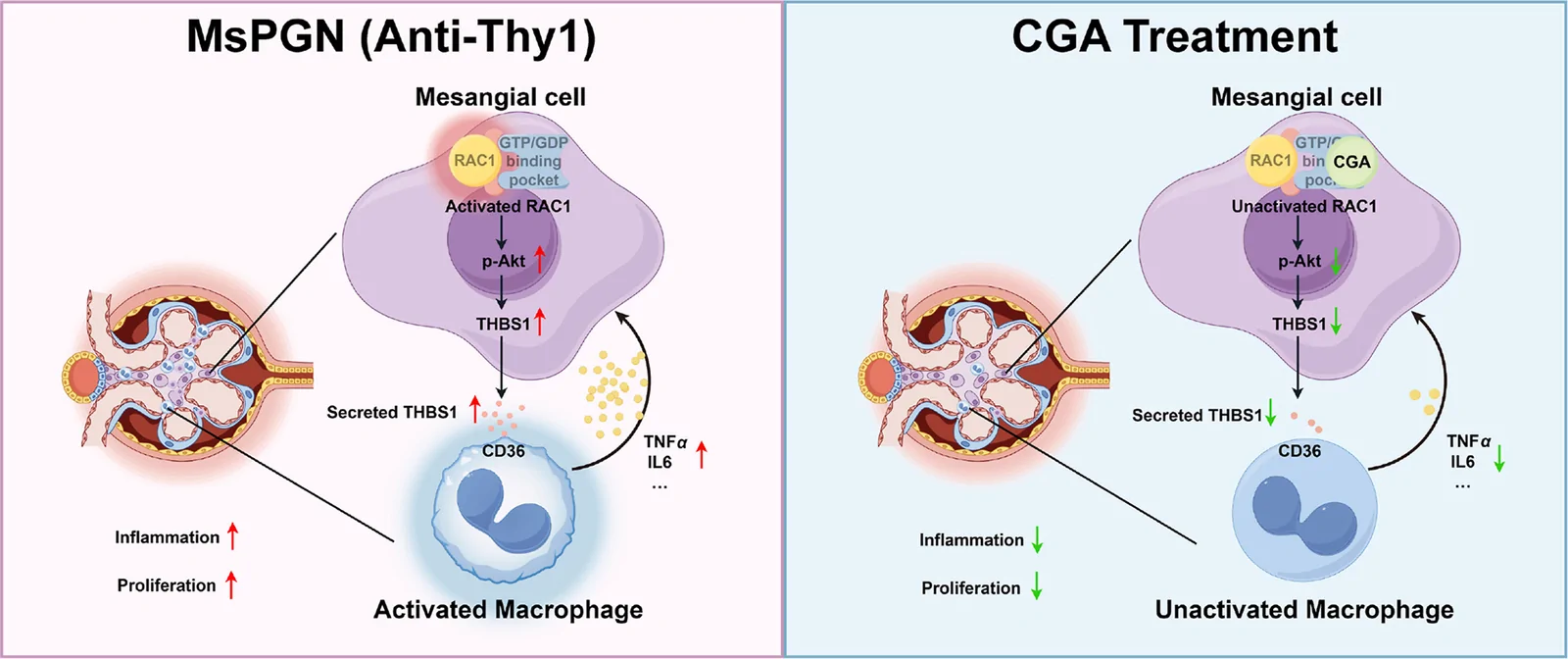

## 1 Introduction

Mesangial cell proliferation is a key pathological feature of various immune-mediated glomerular disorders. Prolonged mesangial cell proliferation can lead to irreversible glomerulosclerosis and progression to end-stage renal disease^1^. Mesangial proliferative glomerulonephritis (MsPGN) is the most common glomerulonephritis. MsPGN accounts for approximately 70.88% of primary glomerular diseases, with IgA nephropathy (IgAN) comprising 45.26% of these patients^2^. Approximately 27% of IgAN patients experience a gradual decline in renal function, leading to end-stage renal disease or a ≥50% reduction in estimated glomerular filtration rate within a decade^3^. The pathogenesis of MsPGN involves complement activation^4^^,^^5^, immune cell infiltration^6^, and the production of pro-inflammatory mediators, resulting in mesangial cell proliferation and increased extracellular matrix deposition^7^^,^^8^. These changes lead to inflammation, glomerular injury, and progressive renal dysfunction. Therefore, inhibiting immune‒inflammatory responses and mesangial cell proliferation is crucial for managing MsPGN. Currently, treatments for MsPGN primarily involve glucocorticoids and cytotoxic agents, which alleviate symptoms but are associated with significant adverse effects. Thus, there is an urgent need to identify novel therapeutic strategies that can inhibit these processes.

Natural products, known for low toxicity and diverse bioactivity, are highly valued in drug discovery. Growing evidence highlights the therapeutic benefits of natural products in treating kidney diseases^9^, ^10^, ^11^, ^12^, ^13^. Chlorogenic acid (CGA), a small molecule compound derived from plants such as honeysuckle^14^, exhibits a broad spectrum of pharmacological properties, including anti-inflammatory, blood glucose regulation, anti-proliferative, anti-fibrotic, and immunomodulatory effects^15^, ^16^, ^17^, ^18^. It has shown efficacy in diabetic nephropathy^19^^,^^20^ and cisplatin-induced kidney injury^21^. Although CGA is well recognized for its anti-inflammatory and anti-proliferative properties by targeting ACAT1, WWP1, and annexin A2^15^^,^^16^^,^^22^, the specific pathways and molecular interactions underlying its beneficial effects in MsPGN are not yet understood. Clarifying these mechanisms is crucial for optimizing CGA's therapeutic potential and developing targeted treatments for MsPGN.

Research has established that thrombospondin 1 (THBS1), a member of the extracellular matrix (ECM) glycoprotein family, plays a critical role in regulating cellular pathophysiological processes, including angiogenesis, proliferation, and apoptosis^23^, ^24^, ^25^, ^26^, ^27^. Upregulation of THBS1 has been observed in kidney tissue of diabetic nephropathy^28^, ischemic renal failure^29^, and other kidney diseases. THBS1 can be regulated by the transcription factor AKT^30^^,^^31^. Additionally, THBS1 acts as a ligand for the integrin membrane protein CD36, expressed on monocytes and macrophages, thereby exerting extensive pro-inflammatory effects^32^, ^33^, ^34^. This cascade may facilitate mesangial cell inflammation and proliferation in MsPGN. Small G proteins are intracellular signaling molecules that act as molecular switches to control various cellular processes, including cytoskeletal remodeling, cell proliferation, and cell migration. Ras-related C3 botulinum toxin substrate 1 (RAC1), a member of the Rho family of small G proteins, activates AKT phosphorylation^35^, ^36^, ^37^, which subsequently upregulates THBS1 expression^38^. Therefore, RAC1-THBS1 targeted therapy may be highly valuable for the treatment of MsPGN. Nevertheless, the regulatory mechanisms by which CGA modulates RAC1-THBS1 remain largely unexplored.

In this study, we investigated the suppressive effects of CGA on inflammation-induced proliferation of glomerular mesangial cells in a model of anti-Thy1 nephritis, which mimics MsPGN and shares a similar pathogenesis^6^^,^^39^, ^40^, ^41^. Mechanistically, by binding to RAC1, CGA can suppress the inflammatory response mediated by THBS1-CD36 interactions and subsequent cellular proliferation.

## 2 Results

### 2.1 CGA alleviates kidney damage in rats with anti-Thy1 nephritis

**Figure 1:**
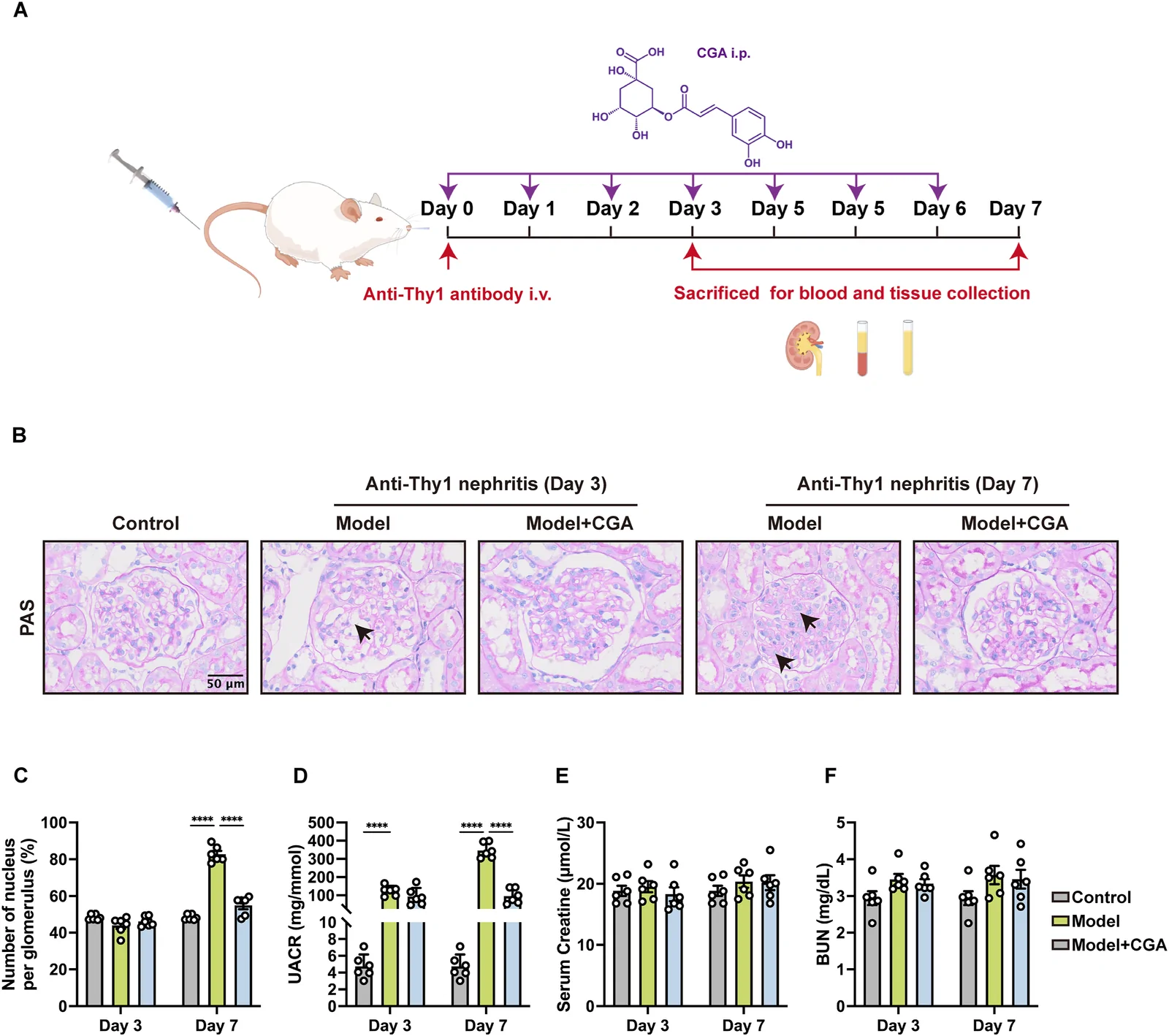
Chlorogenic acid alleviates kidney damage in rats with anti-Thy1 nephritis. (A) Outline of the drug administration protocol for anti-Thy1 rats. Male rats aged 6 weeks were administered intraperitoneal injections of normal saline and 10 mg/kg chlorogenic acid (CGA) for either 3 or 7 days and were then euthanized at the respective time points. (B) Representative images of kidney tissues with periodic acid-Schiff (PAS) staining (Scale bar = 50 μm). (C) Quantification of the number of nuclei per glomerulus after drug treatment (mean ± SEM, *n* = 6, two-way ANOVA, ∗∗∗∗*P* < 0.0001). (D) Urinary albumin-to-creatinine ratios (UACR) of spot urine collected during the study are shown (mean ± SEM, *n* = 6, two-way ANOVA, ∗∗∗∗*P* < 0.0001). (E) Serum creatinine and (F) blood urea nitrogen (BUN) were measured (mean ± SEM, *n* = 6, two-way ANOVA, *P* > 0.05).

To examine the protective properties of CGA *in vivo*, we administered the anti-Thy1 antibody intravenously through the tail vein of rats to induce an anti-Thy1 nephritis model (Fig. 1A). The optimal dosage of CGA for treating anti-Thy1 nephritis was determined through preliminary investigation (Supporting Information Fig. S1A‒S1C). Furthermore, safety assessment confirmed that CGA exhibits no hepatotoxicity or nephrotoxicity, as evidenced by the absence of renal injury in the control rats across all time points (Fig. S1D–S1I). As shown in Fig. 1B and C, CGA treatment significantly reduced the number of nucleated cells in the glomeruli on Day 7 after model induction. The urinary albumin-to-creatinine ratio (UACR) was markedly elevated in model rats on Days 3 and 7 compared with controls, and this increase was effectively attenuated by CGA treatment by Day 7 (Fig. 1D). However, no statistically significant differences were observed in serum creatinine and blood urea nitrogen (BUN) levels among the groups (Fig. 1E and F), consistent with prior findings in studies using the anti-Thy1 nephritis model^40^^,^^42^.

### 2.2 CGA attenuates inflammation and proliferation in anti-Thy1 nephritis

**Figure 2:**
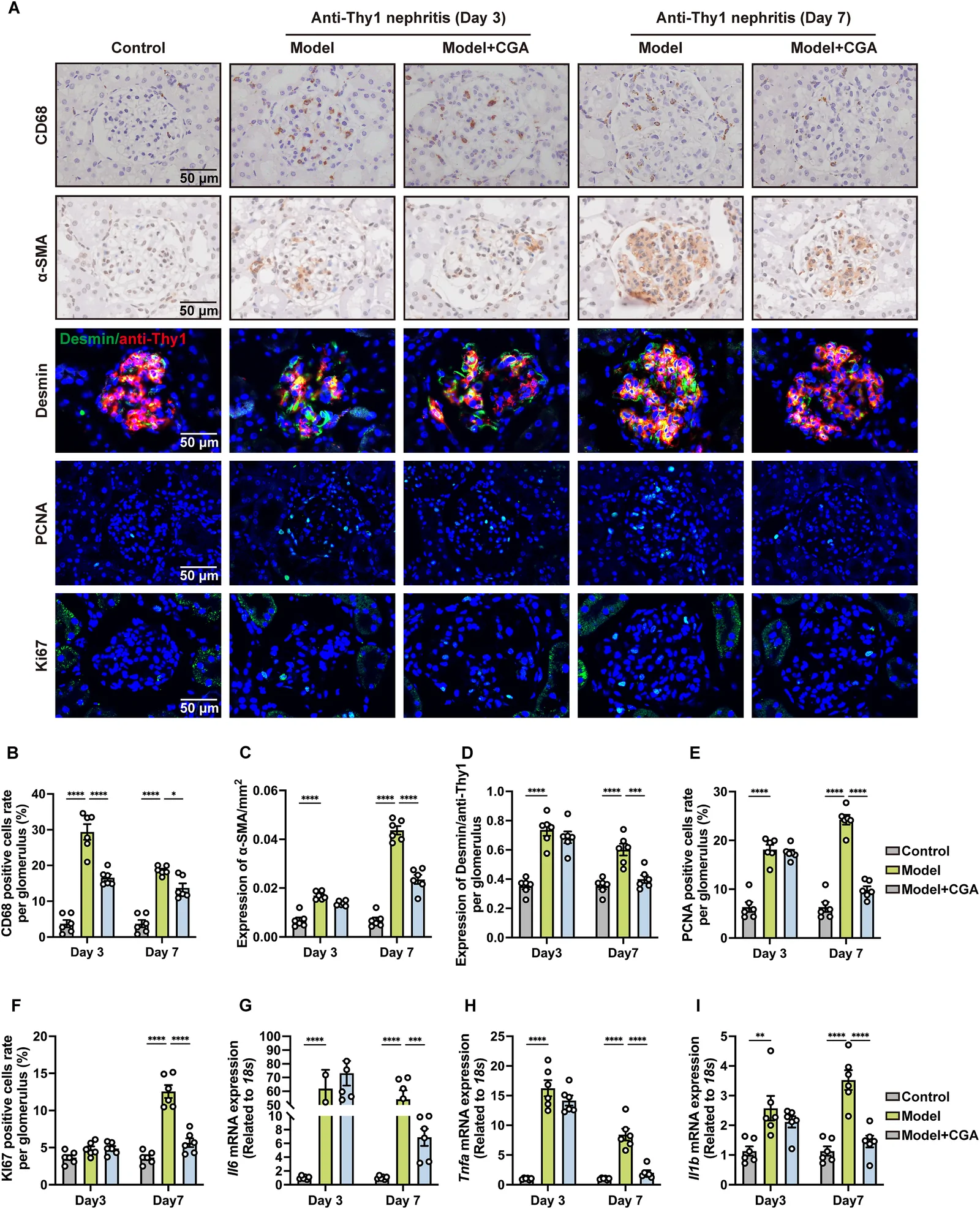
CGA attenuates inflammation and proliferation in anti-Thy1 nephritis. (A) Representative images of CD68, *α*-Smooth Muscle Actin (*α*-SMA), Desmin (blue fluorescence indicates DAPI, green fluorescence indicates Desmin, and red fluorescence indicates anti-Thy1), proliferating cell nuclear antigen (PCNA), and KI67 expression in kidney tissues after drug treatment (Scale bar = 50 μm). Quantification of (B) CD68, (C) *α*-SMA, (D) Desmin (green fluorescence represents Desmin, red fluorescence represents the mesangial cell-specific marker anti-Thy1, Desmin/anti-Thy1 fluorescence ratio quantifies Desmin expression), (E) PCNA and (F) KI67 expression after drug treatment (mean ± SEM, *n* = 6, two-way ANOVA, ∗*P* < 0.05, ∗∗∗∗*P* < 0.0001). mRNA expression levels of (G) *Il6*, (H) *Tnfa*, and (I) *Il1b* were detected using quantitative real-time polymerase chain reaction (qRT-PCR) (mean ± SEM, *n* = 6, two-way ANOVA, ∗∗*P* < 0.01, ∗∗∗∗*P* < 0.0001).

CD68-positive cells were notably present in the glomeruli on Day 3 in the anti-Thy1 nephritis model. Although the number of CD68-positive cells decreased by Day 7, it remained significantly higher than that in the control group (Fig. 2A and B). Moreover, the model group showed a significant increase in mRNA expression of the inflammatory factors *Il6*, *Tnfa*, and *Il1b* on Days 3 and 7, with peak expression on Day 3 (Fig. 2G‒I). In the CGA group, there was a significant reduction in CD68-positive cells within the glomeruli on Day 7 (Fig. 2A and B), accompanied by a marked decrease in *Tnfa* and *Il6* mRNA levels (Fig. 2G‒I). *α*-Smooth muscle actin (*α*-SMA)^43^, ^44^, ^45^, ^46^ and Desmin^47^ are markers of mesangial activation, while proliferating cell nuclear antigen (PCNA)^48^^,^^49^ and KI67^50^ indicate mesangial proliferation. Our research showed that CGA treatment significantly reduced *α*-SMA and Desmin expression (Desmin/anti-Thy1) and decreased the percentage of PCNA and KI67-positive cells in the glomeruli on Day 7 in anti-Thy1 nephritis (Fig. 2A, C‒F). These findings align with the progression profile of anti-Thy1 nephritis, which typically involves an initial surge in immune cell infiltration and inflammatory factor release on Day 3, followed by prominent mesangial proliferation on Day 7. Collectively, these results suggest that CGA may mitigate the inflammatory response, suppress mesangial activation and proliferation in rats with anti-Thy1 nephritis.

### 2.3 AKT/THBS1 is a key pathway involving the therapeutic effects of CGA in anti-Thy1 nephritis

**Figure 3:**
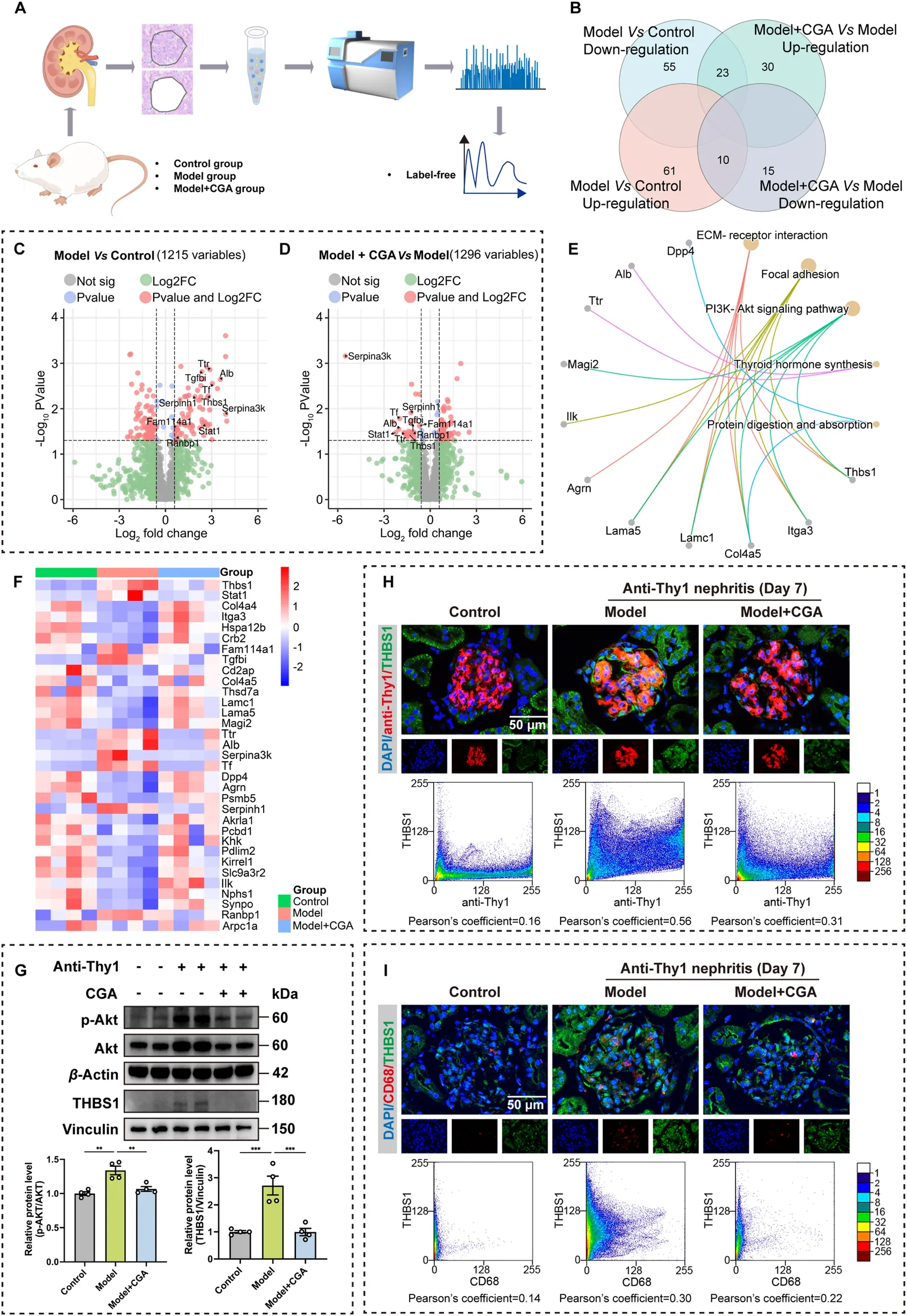
AKT**/**Thrombospondin-1 is a key pathway involving the therapeutic effects of CGA in anti-Thy1 nephritis. (A) Schematic diagram of the method for glomerular laser micro-cutting proteomics. (B) Venn diagram of differently expressed proteins shared by the two comparison groups. Volcano plot of differentially expressed proteins (C) between the model and control groups and (D) between the model + CGA and model groups. (E) Kyoto Encyclopedia of Genes analysis of the commonly expressed proteins between the groups. (F) Heatmap of differently expressed proteins shared by the two comparison groups. (G) *p*-AKT and Thrombospondin-1 (THBS1) expressions in anti-Thy1 nephritis rats were detected by Western blotting (WB) (mean ± SEM, *n* = 4, one-way ANOVA, ∗∗*P* < 0.01, ∗∗∗*P* < 0.001). (H) Representative images of co-staining for THBS1 and the mesangial cell marker anti-Thy1 in glomeruli of anti-Thy1 nephritis rats (blue fluorescence indicates DAPI, green fluorescence indicates THBS1, and red fluorescence indicates anti-Thy1) and fluorescence co-localization analysis. (I) Representative images of co-staining for THBS1 and the macrophage marker CD68 in glomeruli of anti-Thy1 nephritis rats (blue fluorescence indicates DAPI, green fluorescence indicates THBS1, and red fluorescence indicates CD68) and fluorescence co-localization analysis.

To explore the molecular mechanism underlying CGA’s therapeutic effects in anti-Thy1 nephritis, we employed laser microdissection proteomics in the glomerulus to analyze protein expression differences among groups after 7 days of modeling (Fig. 3A). Differentially expressed proteins were identified based on criteria of ∣logFC∣ > 0.58 and *P* < 0.05. A total of 149 proteins were differentially expressed between the model and control groups, with 71 upregulated and 78 downregulated (Fig. 3B and C). The CGA group exhibited increased expression of 53 proteins and decreased expression of 25 proteins compared to the model group (Fig. 3B–D). Following CGA treatment, compared with the control group, the expression of 33 changed proteins in the model group was reversed (Fig. 3B), such as THBS1, STAT1, Serpina3k, etc. Among proteins exhibiting upregulated expression in the model group and downregulation after CGA treatment, PCR validation of the top 10 fold change (∣logFC∣)-ranked proteins (Supporting Information Fig. S2B‒S2K) demonstrated concordance with proteomic data, further validating the reliability of the proteomics findings.

The majority of the biological processes associated with the identified proteins were linked to kidney diseases, including extracellular matrix organization, renal filtration, and actin cytoskeleton organization (Fig. S2A). Kyoto Encyclopedia of Genes and Genomes (KEGG) pathway analysis revealed significant enrichment in pathways, including ECM-receptor interaction, focal adhesion, phosphoinositide 3-kinase (PI3K)–AKT signaling, thyroid hormone synthesis, and protein digestion and absorption (Fig. 3E). The PI3K–AKT pathway is involved in both inflammatory responses and cell proliferation, with PI3K-dependent AKT activation driving proliferative changes in glomerular mesangial cells in anti-Thy1 nephritis^39^. Previous studies employing inflammatory factor chip technology and network pharmacology identified the PI3K-AKT pathway^40^ as critical in treating anti-Thy1 nephritis with Shenhua tablets. LC/ESI‒MS and GC/EI‒MS assays identified CGA as a significant component of the Shenhua tablet^51^, prompting further investigation into the PI3K-AKT pathway. Furthermore, THBS1, a protein enriched in the PI3K-AKT pathway (Fig. 3E and F), can interact with the CD36 receptor on macrophages^32^, triggering macrophage activation and the subsequent secretion of pro-inflammatory factors^33^, which further promotes mesangial cell activation and proliferation. THBS1 also exerts a chemotactic effect on monocytes, facilitating macrophage migration^52^. Subsequent experimental investigations of AKT and THBS1, pivotal proteins in the PI3K–AKT pathway, were conducted. Immunofluorescence results showed increased THBS1 expression in the glomeruli of anti-Thy1 nephritis rats, which was reduced after CGA treatment in a dose-dependent manner (Fig. S1A). Western blotting (WB) analysis showed a significant decrease in AKT and THBS1 expression in the glomeruli following CGA treatment (Fig. 3G, Fig. S2L and S2M). To validate the proteomic findings and investigate CGA's potential to inhibit mesangial cell proliferation by regulating THBS1, immunofluorescence co-staining was used to assess THBS1 expression levels and localization in anti-Thy1 nephritis. Results indicated a reduction in THBS1 expression in the mesangial area following CGA treatment (Fig. 3H, I, Fig. S2N‒S2P). To further confirm the cellular origin of THBS1, co-localization analysis was conducted using immunofluorescence co-staining for THBS1 alongside the mesangial cell marker anti-Thy1 and the macrophage marker CD68. This analysis demonstrated specific co-localization of THBS1 with mesangial cells (Pearson's coefficient >0.5), whereas co-localization with macrophages was limited. These findings indicate that THBS1 primarily originates from mesangial cells (Fig. S2O and S2P). These findings support that the AKT/THBS1 pathway plays a crucial role in CGA's therapeutic effects in anti-Thy1 nephritis. Notably, in the B cell-activating factor (BAFF) mouse model, which mimics autoimmune diseases, we observed significant increases in serum creatinine, urinary protein, the number of glomerular nuclei, the number of PCNA-positive cells, the number of CD68-positive macrophages, and THBS1 expression in the kidneys of mice with mesangial proliferation (Supporting Information Fig. S3A‒S3G). These findings further support the hypothesis that upregulated THBS1 is closely associated with inflammation and glomerular mesangial proliferation. Additionally, previous studies have demonstrated that THBS1 plays a critical role in the progression of renal fibrotic disease^53^^,^^54^. To evaluate whether THBS1 inhibition ameliorates fibrosis, we performed Masson's trichrome staining and WB analysis for fibrosis markers Collagen I and Vimentin in rat glomeruli at Day 7 post-model induction. Results revealed that CGA-mediated suppression of THBS1 significantly attenuated glomerular fibrosis, as evidenced by reduced collagen deposition on trichrome staining and decreased expression of Collagen I and Vimentin (Fig. S3H‒S3M).

### 2.4 CGA directly binds to RAC1 and inhibits its activity

**Figure 4:**
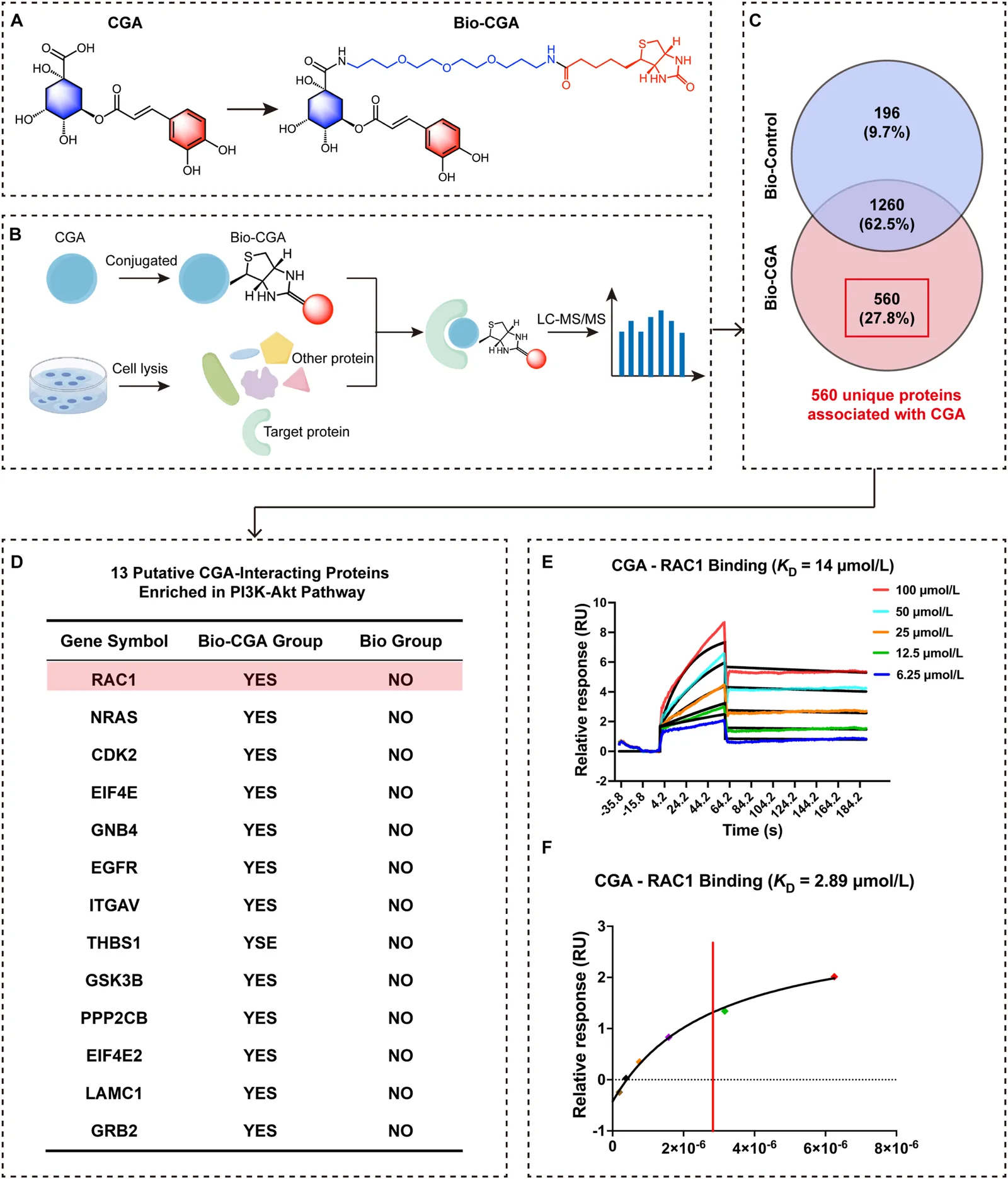
CGA directly binds to Ras-related C3 botulinum toxin substrate 1. (A) Schematic depiction of labeling CGA with biotin. (B) A schematic representation of the streptavidin-biotin affinity pull-down assay. (C) Venn diagram of CGA and HRMCs' direct binding protein. (D) 13 putative CGA-interacting proteins enriched in the phosphoinositide 3-kinase (PI3K)-AKT pathway. (E) Binding kinetic curve of CGA to Ras-related C3 botulinum toxin substrate 1 (RAC1). (F) Steady-state fitting plot of CGA with RAC1.

We further elucidate the mechanisms by which CGA exerts anti-inflammatory and antiproliferative effects by regulating the AKT/THBS1 pathway. Biotinylated CGA (Bio-CGA) was synthesized (Fig. 4A), followed by incubation of human renal mesangial cells (HRMCs) with Bio-CGA and a streptavidin-biotin affinity pull-down assay to identify precipitated proteins *via* mass spectrometry analysis (Fig. 4B, Supporting Information Table S2). A total of 560 unique proteins associated with CGA were identified (Fig. 4C). Thirteen of these proteins were enriched in the PI3K–AKT pathway according to KEGG enrichment analysis (Fig. 4D). Each protein was then docked with CGA, revealing that RAC1 exhibited the highest binding affinity (Supporting Information Fig. S4C‒S4P). Further literature review confirmed that aberrant activation of RAC1 plays a central role in the pathogenesis of MsPGN and experimental anti-Thy1 nephritis, directly regulating mesangial cell proliferation and inflammatory responses^55^, ^56^, ^57^. In contrast, none of the other candidate proteins have been clearly linked to these disease mechanisms, lacking direct “target–mechanism” evidence. Based on this target prioritization analysis and mechanistic relevance, we focused subsequent validation on RAC1. To validate the direct binding between CGA and RAC1, surface plasmon resonance analysis was performed. Results demonstrated concentration-dependent binding of CGA to RAC1, with kinetic analysis yielding an equilibrium dissociation constant (*K*_D_) of 14 μmol/L (Fig. 4E). Direct equilibrium measurements yielded a *K*_D_ of 2.89 μmol/L (Fig. 4F). These data indicate high-affinity binding between CGA and RAC1.

**Figure 5:**
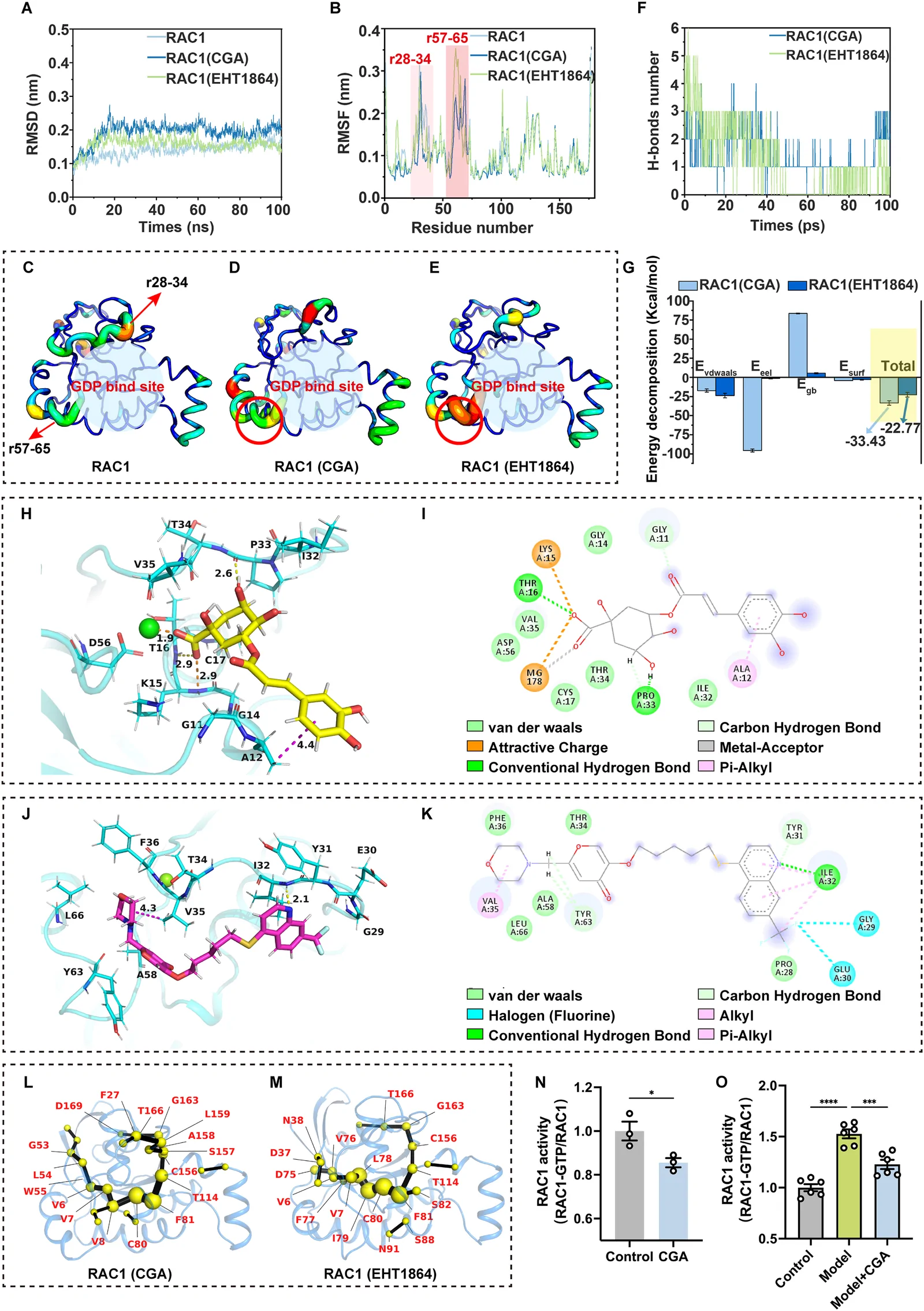
CGA inhibits RAC1 activation by binding to its GTP/GDP-binding domain. (A) RMSD plots of the RAC1‒CGA complex and RAC1‒EHT1864 complex. (B) RMSF plots of the RAC1‒CGA complex and RAC1‒EHT1864 complex. (C) B-factor plot of RAC1. (D) B-factor plot of RAC1 in the CGA-bound state. (E) B-factor plot of RAC1 in the EHT1864-bound state. (F) Number of hydrogen bonds formed between CGA and RAC1, and between EHT1864 and RAC1. (G) Binding free energy and energy decomposition diagrams for CGA-RAC1 and EHT1864-RAC1 interactions. (H) and (I) 3D and 2D interaction diagrams of CGA with RAC1, respectively. (J) and (K) 3D and 2D interaction diagrams of EHT1864 with RAC1, respectively. (L) Shortest path analysis of internal interactions in the CGA-RAC1 complex. (M) Shortest path analysis of internal interactions in the EHT1864-RAC1 complex. (N) Detection of the effect of CGA on RAC1 activity in HRMCs (mean ± SEM, *n* = 3, one-way ANOVA, ∗*P* < 0.05). (O) Detection of the effect of CGA on RAC1 activity in glomeruli of anti-Thy1 nephritis rats on Day 7 of the model (mean ± SEM, *n* = 6, one-way ANOVA, ∗∗∗*P* < 0.001, ∗∗∗∗*P* < 0.0001). The activity of RAC1 was measured by detecting the ratio of RAC1-GTP/RAC1 using G-LISA assays.

To elucidate the binding mode of CGA to RAC1, molecular dynamics (MD) simulations were performed on three systems: (1) CGA‒RAC1 complex, (2) EHT1864-RAC1 complex, and (3) unbound RAC1 protein. Each system underwent 100-ns MD simulations to characterize the molecular mechanism of CGA‒RAC1 interaction. EHT1864—a reported competitive inhibitor of RAC1 targeting the GTP/GDP-binding domain—served as a positive control to analyze ligand-induced conformational dynamics. Root-mean-square deviation (RMSD) analysis revealed that all systems reached equilibrium after 20 ns, with stable fluctuations in RMSD throughout the simulation (Fig. 5A). Compared to unbound RAC1, both ligands increased protein flexibility, with CGA inducing greater conformational displacement. Root-mean-square fluctuation (RMSF) analysis identified residues 28‒34 and 57‒65 as key flexible regions in ligand-bound states (Fig. 5B and C), both of which structurally support the GDP-binding site. While maintaining rigidity in other domains (Fig. 5C‒E), both ligands specifically enhanced flexibility in residues 57‒65 (switch II region). Interaction analysis revealed comparable hydrogen bonding between RAC1 and either ligand (Fig. 5F).

Molecular docking results demonstrated that CGA forms hydrogen bonds with Pro33 and Thr16 of RAC1, while engaging in electrostatic interactions with Lys15 and the Mg^2+^ ion (Fig. 5H and I). Notably, Ile32, Thr34, and Val35 participate in both CGA and EHT1864 binding to RAC1 through van der Waals and hydrophobic interactions (Fig. 5H‒K). Allosteric motion analysis revealed analogous impacts of CGA and EHT1864 on RAC1 residue dynamics. CGA binding induced anti-correlated motions between the r1–36 and r65–71 domains, while other regions exhibited dynamic patterns highly similar to the EHT1864-bound state (Supporting Information Fig. S5A and S5B). Shortest Path Map (SPM) analysis further demonstrated remarkable conservation of core interaction pathways in RAC1 upon ligand binding, with Ile79, Cys80, and Phe81 identified as critical nodes (Fig. 5L and M). These structural findings indicate that CGA modulates RAC1 conformation through residue interactions and allosteric motions, similar to EHT1864, suggesting potential inhibition of RAC1 activity *via* competitive binding to the GDP/GTP domain. To identify the critical residues involved in CGA binding to RAC1, we performed site-directed mutagenesis to alanine on a basic residue (LYS15), a neutral nonpolar residue (PRO33), and a neutral polar residue (THR34), selected based on their distinct chemical properties. As shown in Fig. S5C–S5E, these mutations abolished the binding of RAC1 to CGA, demonstrating that LYS15, PRO33, and THR34 are key amino acids mediating the CGA–RAC1 interaction.

To directly assess functional consequences, RAC1 activation status was quantified using G-LISA, expressed as the RAC1-GTP/RAC1 ratio. As shown in Fig. 5N–O, CGA significantly reduced RAC1-GTP/RAC1 ratios in HRMCs and glomeruli of the model group, confirming suppression of RAC1 activity. THBS1, identified in the proteomic screening, was also detected in the pull-down experiment. Still, previous results have shown dynamic changes in THBS1 protein and mRNA levels in glomeruli pre- and post-CGA treatment (Fig. 3G, H, Fig. S2G), suggesting an indirect association with CGA rather than direct binding. The findings indicate that CGA exerts its inhibitory effect on RAC1 activity by competitively binding to the active pocket of RAC1.

### 2.5 CGA attenuates mesangial cell proliferation by suppressing RAC1 activation and concomitantly attenuating THBS1 expression

To determine the optimal concentration of CGA for evaluating its anti-inflammatory and antiproliferative effects, we assessed CGA cytotoxicity in HRMCs (Supporting Information Fig. S6A). TNF*α*, a well-characterized inflammatory cytokine known to stimulate RAC1 activation^58^^,^^59^ and upregulate THBS1 expression^31^^,^^60^^,^^61^, was selected to simulate inflammation-induced proliferation *in vitro*. Based on TNF*α*-stimulated CCK8 proliferation assays and quantitative real-time polymerase chain reaction (qRT-PCR) analyses of KI67 and PCNA expression (Fig. S6B–S6D), we selected 140 μmol/L CGA for subsequent experiments.

**Figure 6:**
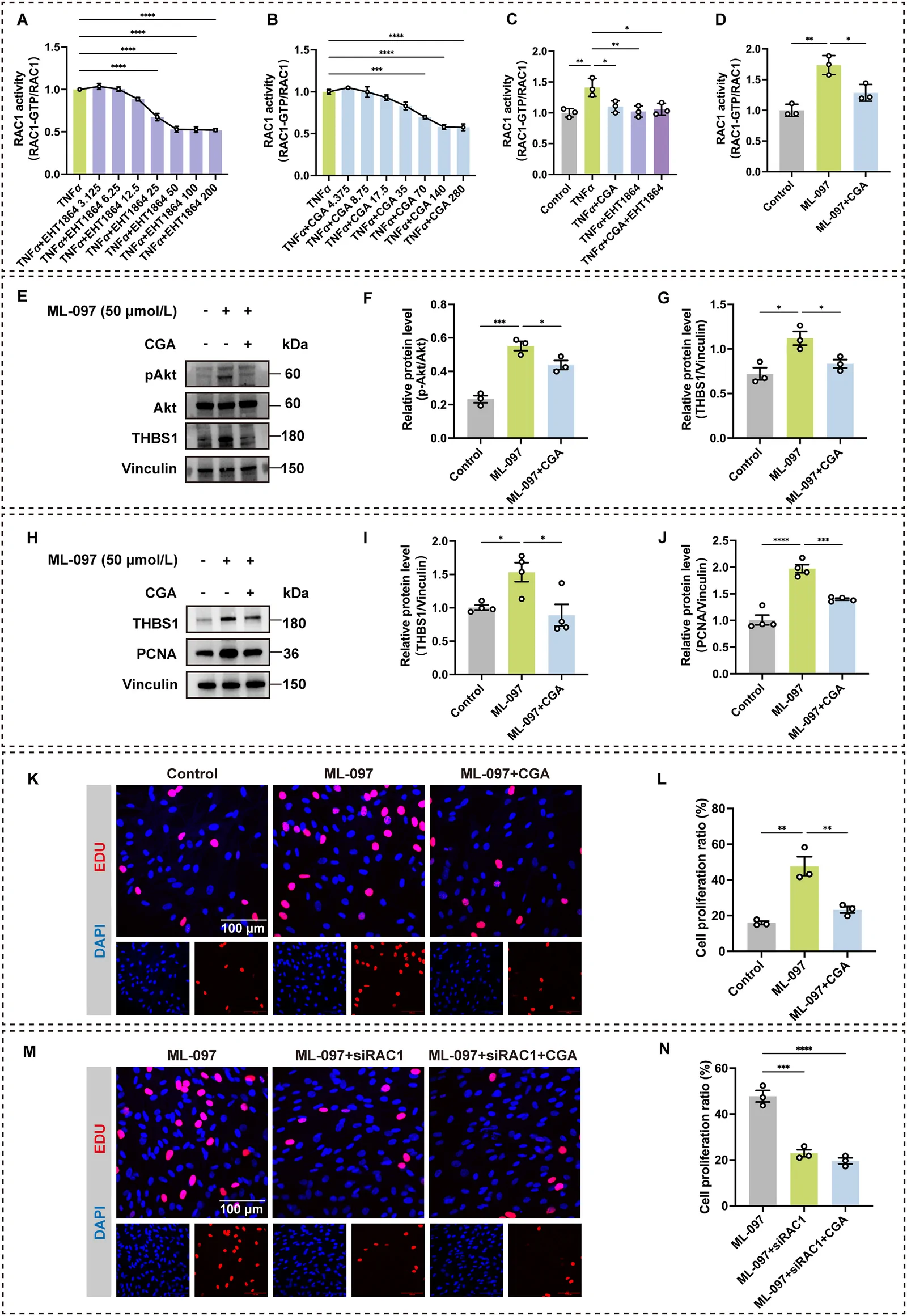
CGA suppresses mesangial cell proliferation by attenuating aberrant RAC1 activation. (A‒C) Detection of the impact of CGA and EHT1864 on TNF*α*-induced RAC1 activity in HRMCs (mean ± SEM, *n* = 3, one-way ANOVA, ∗*P* < 0.05, ∗∗*P* < 0.01, ∗∗∗*P* < 0.001, ∗∗∗∗*P* < 0.0001). (D) Detection of the impact of CGA on RAC1 activator ML-097-induced RAC1 activity in HRMCs (mean ± SEM, *n* = 3, one-way ANOVA, ∗*P* < 0.05, ∗∗*P* < 0.01). (E‒G) WB analysis of *p*-AKT and THBS1 protein expressions in HRMCs stimulated with the RAC1 activator ML-097 (mean ± SEM, *n* = 3, one-way ANOVA, ∗*P* < 0.05, ∗∗∗*P* < 0.001). (H‒J) WB analysis of THBS1 and PCNA protein expressions in HRMCs stimulated with the RAC1 activator ML-097 (mean ± SEM, n = 4, one-way ANOVA, ∗*P* < 0.05, ∗∗∗*P* < 0.001, ∗∗∗∗*P* < 0.0001). (K‒L) Effects of CGA in HRMCs proliferation after ML-097 stimulation detected by EDU assay (Scale bar = 100 μm, mean ± SEM, *n* = 3, one-way ANOVA, ∗∗*P* < 0.01). (M‒N) Effects of CGA in HRMCs proliferation after ML-097 knockdown detected by EDU assay (Scale bar = 100 μm, mean ± SEM, *n* = 3, one-way ANOVA, ∗∗∗*P* < 0.001, ∗∗∗∗*P* < 0.0001).

Establishing the role of RAC1 in mesangial cell proliferation. Under TNF*α* stimulation, RAC1 activity increased significantly, whereas treatment with CGA or the RAC1-specific inhibitor EHT1864 alone reduced RAC1 activity. Notably, no additive inhibitory effects were observed when CGA and EHT1864 were combined (Fig. 6A‒C). Of note, RAC1 overexpression did not induce changes in RAC1 enzymatic activity (Fig. S6E). Using ML-097 (50 μmol/L), a validated RAC1 activator, we investigated whether CGA suppresses THBS1 expression and cell proliferation by inhibiting RAC1 signaling. Compared to ML-097-stimulated controls (50 μmol/L), CGA-treated groups (140 μmol/L) exhibited significant reductions in *p*-AKT, THBS1, and PCNA protein levels, as well as a decrease in EDU + proliferating cell counts (Fig. 6D‒L). Furthermore, after RAC1 knockdown *in vitro*, the number of EDU + cells decreased, and CGA treatment could not further reduce EDU + cell counts (Fig. 6M and N). This suggests that RAC1 knockdown eliminates CGA's antiproliferative effects. In summary, CGA's therapeutic effects are mainly dependent on the RAC1 signaling pathway, and RAC1 knockdown *in vitro* abolishes CGA's therapeutic effects.

**Figure 7:**
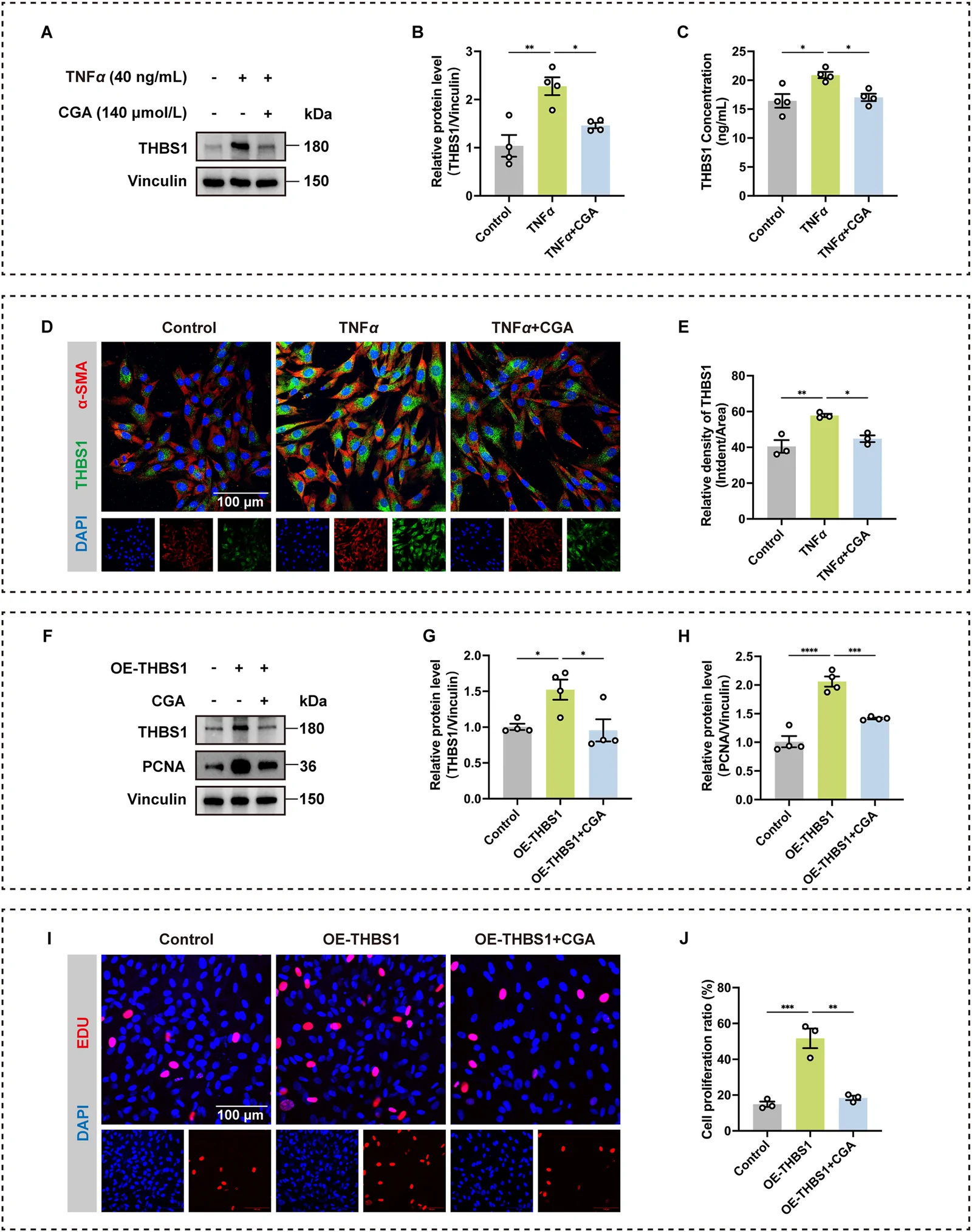
CGA suppresses mesangial cell proliferation by attenuating THBS1 expression. (A, B) WB analysis of THBS1 protein expression in HRMCs following TNF*α* stimulation (mean ± SEM, *n* = 4, one-way ANOVA, ∗*P* < 0.05, ∗∗*P* < 0.01). (C) Detection of THBS1 levels in mesangial cell supernatants by ELISA (mean ± SEM, n = 4, one-way ANOVA, ∗*P* < 0.05). (D, E) Illustrative images and statistical data depicting the THBS1 within HRMCs following TNF*α* stimulation (Scale bar = 100 μm, mean ± SEM, *n* = 3, one-way ANOVA, ∗*P* < 0.05, ∗∗*P* < 0.01). (F‒H) WB analysis of THBS1 and PCNA protein expressions in HRMCs following THBS1 overexpression (THBS1-OE) (mean ± SEM, *n* = 4, one-way ANOVA, ∗*P* < 0.05, ∗∗∗*P* < 0.001, ∗∗∗∗*P* < 0.0001). (I, J) Effects of CGA in HRMCs following THBS1 overexpression (THBS1-OE) detected by EDU assay (Scale bar = 100 μm, mean ± SEM, *n* = 3, one-way ANOVA, ∗∗*P* < 0.01, ∗∗∗*P* < 0.001).

Elucidating the role of THBS1 in mesangial cell proliferation. As shown in Fig. 7A‒E and Fig. S6F‒S6H, CGA significantly suppressed both THBS1 expression and secretion under inflammatory conditions, with 140 μmol/L CGA exerting the most potent suppressive effect. To determine whether CGA inhibits proliferation *via* THBS1 suppression, we performed THBS1 overexpression experiments. Compared to the THBS1-overexpressing group, CGA treatment (140 μmol/L) reduced THBS1 and PCNA expression levels (Fig. 7F and G).

Collectively, these findings demonstrate that CGA inhibits TNF*α*-induced mesangial cell proliferation by suppressing aberrant RAC1 activation and reducing THBS1 expression.

### 2.6 CGA restrains AKT phosphorylation and THBS1 expression by targeting RAC1

**Figure 8:**
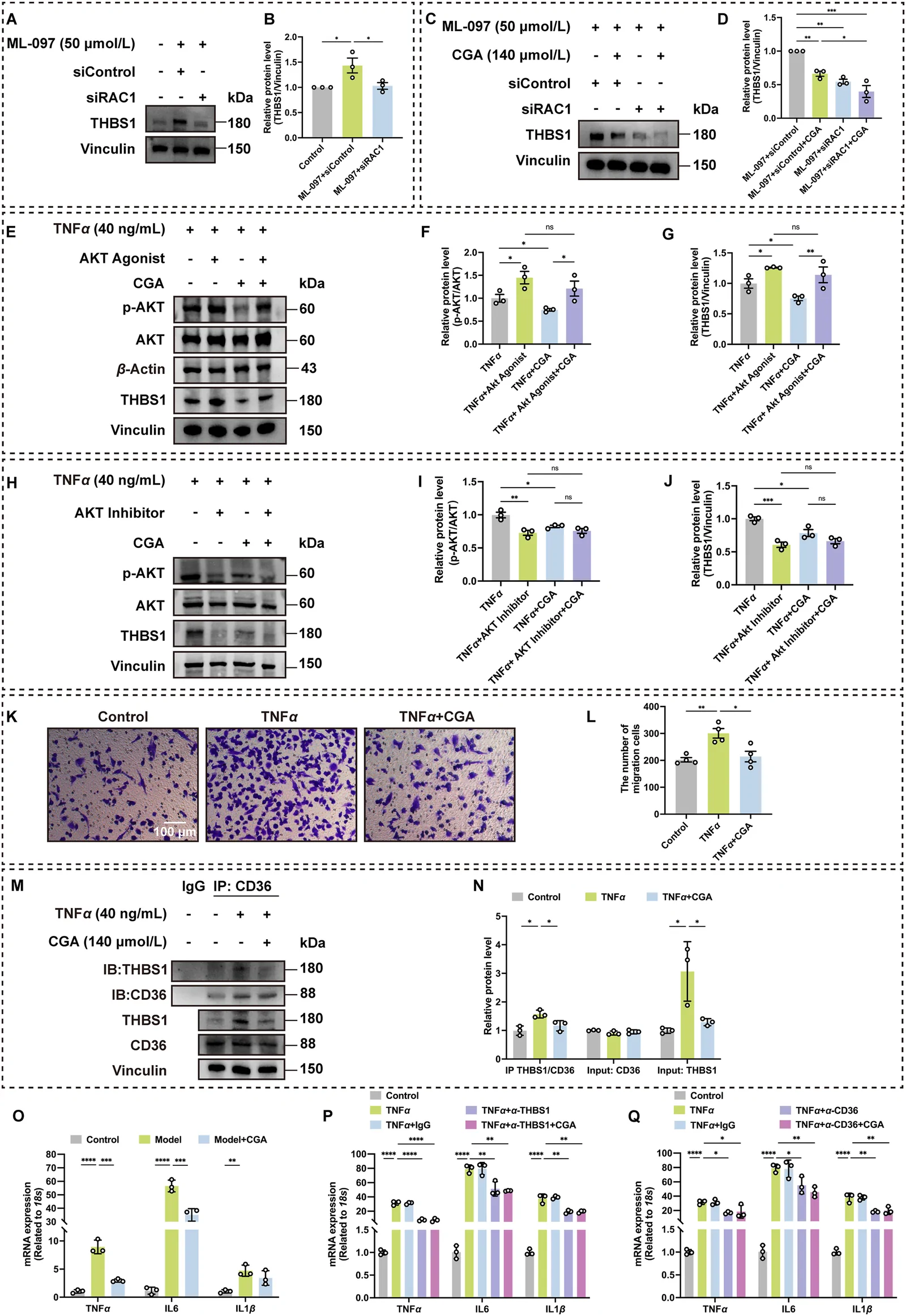
CGA modulates macrophage activation *via* the RAC1-AKT-THBS1-CD36 axis. (A, B) WB detected the impact of activator ML-097 on THBS1 protein expression levels in HRMCs after RAC1 knockdown (mean ± SEM, *n* = 3, one-way ANOVA, ∗*P* < 0.05). (C, D) WB detected the impact of CGA on THBS1 protein expression levels in HRMCs after RAC1 knockdown (mean ± SEM, *n* = 3, one-way ANOVA, ∗*P* < 0.05, ∗∗*P* < 0.01, ∗∗∗*P* < 0.001). (E‒G) *p*-AKT and THBS1 protein expressions in HRMCs were detected after using an AKT agonist in the inflammatory state (mean ± SEM, *n* = 3, one-way ANOVA, ∗*P* < 0.05, ∗∗*P* < 0.01, ∗∗∗*P* < 0.001, ns no significance). (H‒J) *p*-AKT and THBS1 protein expressions in HRMCs were detected after using an AKT inhibitor in the inflammatory state (mean ± SEM, *n* = 3, one-way ANOVA, ∗*P* < 0.05, ∗∗*P* < 0.01, ∗∗∗*P* < 0.001, ns, no significance). (K, L) Crystal violet staining assay for the effect of HRMCs on macrophage migration ability under inflammatory conditions (mean ± SEM, *n* = 4, one-way ANOVA, ∗*P* < 0.05, ∗∗*P* < 0.01). (M, N) Co-IP assay for the binding ability of THBS1 secreted by HRMCs under inflammatory conditions to the CD36 receptor on macrophage surfaces (mean ± SEM, *n* = 3, one-way ANOVA, ∗*P* < 0.05). (O) The level of inflammatory factors in macrophages is observed when co-cultured with HRMCs in an inflammatory state (mean ± SEM, *n* = 3, one-way ANOVA, ∗∗*P* < 0.01, ∗∗∗*P* < 0.001, ∗∗∗∗*P* < 0.0001). (P, Q) Attenuation of macrophage inflammatory cytokine secretion by THBS1/CD36-neutralizing antibodies in co-culture systems (mean ± SEM, *n* = 3, one-way ANOVA, ∗*P* < 0.05, ∗∗*P* < 0.01, ∗∗∗∗*P* < 0.0001).

To validate CGA's inhibition of THBS1 *via* RAC1 regulation, we used siRNA-mediated RAC1 knockdown in HRMCs. Results demonstrated that upon RAC1 downregulation, ML-097 did not induce THBS1 expression (Fig. 8A and B), confirming that CGA's suppressive effect on THBS1 was mediated by RAC1 (Fig. 8C and D). Under TNF*α* simulation, the AKT agonist (SC79) increased AKT phosphorylation and THBS1 levels, negating CGA's therapeutic effects (Fig. 8E‒G). AKT inhibitor (AKT inhibitor VIII) reduced AKT phosphorylation and THBS1 levels, and CGA exhibited no additional inhibitory effects on phosphorylation or expression (Fig. 8H‒J). Previous studies have shown that RAC1 does not directly regulate AKT phosphorylation, but rather modulates downstream cellular functions by interacting with other signaling molecules involved in AKT phosphorylation^62^, ^63^, ^64^. It has been reported that aberrant activation of RAC1 enables it to bind PI3K 110*β*, thereby participating in the regulation of AKT phosphorylation^65^. To validate the interaction between RAC1 and PI3K 110*β*, we conducted co-immunoprecipitation assays. As shown in Supporting Information Fig. S7A and S7B, treatment with the RAC1 agonist ML-097 significantly enhanced the binding between RAC1 and PI3K 110*β*.

These findings demonstrate that CGA effectively inhibits RAC1 activity and suppresses AKT phosphorylation, significantly downregulating THBS1 expression under inflammatory conditions.

### 2.7 CGA suppresses the inflammatory response mediated by THBS1‒CD36 interactions

THBS1 binds to the CD36 receptor on macrophages, activating them and promoting the secretion of inflammatory factors, leading to mesangial cell activation and proliferation. We first confirmed elevated THBS1 secretion in HRMCs supernatants under inflammatory conditions using ELISA (Fig. 7C). Co-culturing macrophages with inflamed HRMCs demonstrated HRMC-induced macrophage chemotaxis (Fig. 8K and L, Fig. S7C). CGA treatment effectively reversed these pathological processes (Fig. 8K and L, Fig. S7C). To confirm the binding of HRMC-secreted THBS1 to macrophage CD36 receptors, we performed Co-IP assays in the co-culture system (Fig. S7D), using CD36 as the immunoprecipitation (IP) antibody to detect THBS1–CD36 interaction. The results showed a significant increase in THBS1 binding to CD36 under inflammatory conditions, which was reduced by CGA treatment (Fig. 8M and N, Fig. S7D). Macrophages were then isolated for qRT-PCR analysis, revealing that inflamed HRMCs induced TNF*α*, IL6, and IL1*β* mRNA expression in macrophages. CGA treatment significantly attenuated macrophage secretion of pro-inflammatory cytokines (Fig. 8O). To delineate the core role of the THBS1‒CD36 axis in CGA-mediated suppression of macrophage activation, functional blockade was performed using neutralizing antibodies against THBS1 or CD36 in an inflammatory co-culture system. As shown in Fig. 8P and Q, under cytokine-primed mesangial cell stimulation, both anti-THBS1 and anti-CD36 antibodies significantly reduced mRNA levels of macrophage activation markers (IL6, IL1*β*, TNF*α*) compared to IgG controls. Notably, co-administration of CGA with either antibody failed to suppress macrophage activation further. These results establish the THBS1–CD36 pathway as the dominant mechanism underlying CGA's anti-inflammatory effects.

### 2.8 CGA exhibits the potential to arrest mesangial cell cycle alterations and proliferation induced by inflammatory factors

**Figure 9:**
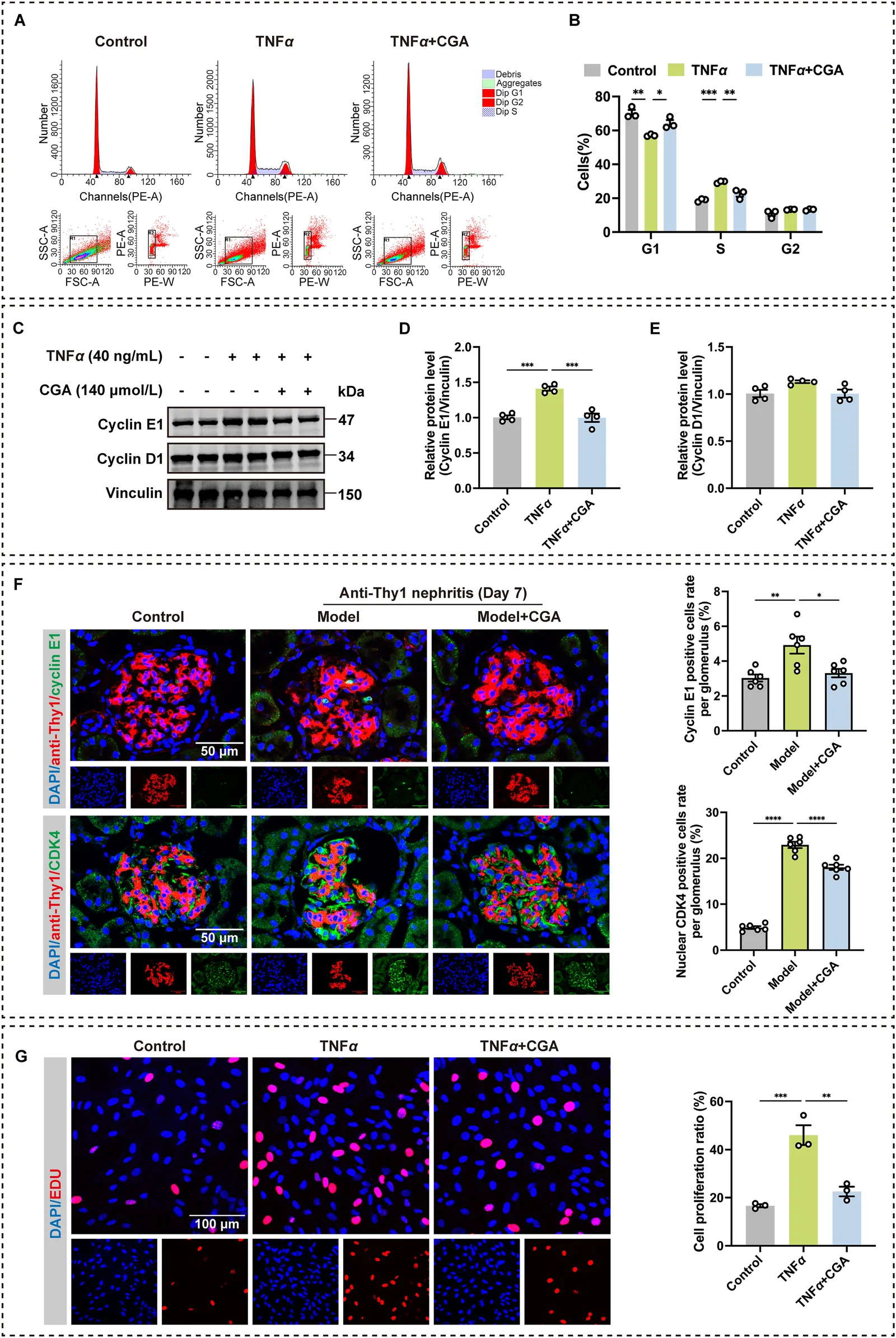
CGA exhibits the potential to arrest mesangial cell cycle alterations and proliferation induced by inflammatory factors. (A, B) Effects of inflammatory factors on HRMCs detected by flow cytometry cell cycle analysis (mean ± SEM, *n* = 3, one-way ANOVA, ∗*P* < 0.05, ∗∗*P* < 0.01, ∗∗∗*P* < 0.001). (C‒E) Cyclin E1 and Cyclin D1 protein expressions within HRMCs were detected by WB (mean ± SEM, *n* = 4, one-way ANOVA, ∗∗∗*P* < 0.001). (F) Illustrative images and statistical data depicting the levels of Cyclin E1 and CDK4 within glomeruli (Scale bar = 50 μm, mean ± SEM, *n* = 6, one-way ANOVA, ∗*P* < 0.05, ∗∗*P* < 0.01, ∗∗∗∗*P* < 0.0001). (G) EDU was used to detect HRMC proliferation (Scale bar = 100 μm, mean ± SEM, *n* = 3, one-way ANOVA, ∗∗*P* < 0.01, ∗∗∗*P* < 0.001).

To evaluate the impact of macrophage-secreted inflammatory factors on HRMCs, we conducted flow cytometry to analyze HRMC cell cycle and employed EDU staining to assess cell proliferation. TNF*α* stimulation facilitated HRMC transition from G1 to S phase, while CGA treatment increased G1 phase duration and reduced S phase progression, indicating G1 phase arrest (Fig. 9A and B). Whole-cell WB and renal glomerular immunofluorescence (IF) findings were consistent with flow cytometry results, showing Cyclin E1 upregulation—governing the G1-to-S phase transition—after TNF*α* stimulation, without changes in Cyclin D1 expression (Fig. 9C‒F). Increased nuclear translocation of CDK4 was also observed (Fig. 9H and I). EDU results further supported CGA's anti-proliferative effect (Fig. 9G).

## 3 Discussion

This research, for the first time, revealed that CGA has therapeutic effects on inflammatory and proliferative responses in MsPGN, by targeting the RAC1–AKT–THBS1–CD36 axis, which is key in inflammation-induced responses and mesangial cell proliferation.

The anti-Thy1 model is a well-established experimental MsPGN model^66^^,^^67^, in which the pathogenic mechanism involves binding of Thy1 antibodies to Thy1 antigens expressed on mesangial cells, which initiates a cascade of immune-mediated inflammatory responses that ultimately result in pathological mesangial cell proliferation^68^^,^^69^. This has been widely used in nephrology research as a valuable tool for elucidating the molecular mechanisms underlying the pathogenesis of mesangial proliferative glomerulonephritis. Our experimental findings from animal studies demonstrate that, in the anti-Thy1 nephritis model, early-phase CD68-positive macrophage infiltration triggers a pro-inflammatory cascade that subsequently induces pathological mesangial cell proliferation. In contrast, administration of CGA effectively reverses this cascade.

To elucidate the precise mechanisms by which CGA modulates aberrant immune and proliferative responses in glomeruli, we conducted a proteomic analysis of glomerular samples. Then, we confirmed that THBS1, enriched in the PI3K–AKT pathway, serves as a pivotal protein in the treatment of anti-Thy1 nephritis. In line with previous research^70^^,^^71^, our work found that THBS1 expression was elevated in the anti-Thy1 nephritis model and subsequently decreased following CGA treatment. Similarly, in BAFF-transgenic IgAN models, which exhibit autoimmune characteristics such as B-cell hyperplasia and hypergammaglobulinemia, significant upregulation of THBS1 was observed in mice with established IgAN, characterized by circulating immune complexes and glomerular immunoglobulin deposits^72^^,^^73^. THBS1 suppression by CGA treatment underscores its pathogenic role in MsPGN, providing mechanistic evidence for CGA's therapeutic efficacy in mesangial proliferative nephropathy. THBS1, a glycoprotein released during tissue injury and remodeling^74^, drives pathogenesis in atherosclerosis^75^ and glomerulonephritis^76^. It promotes inflammation through monocyte chemotaxis and immune dysregulation^33^, functioning as a ligand for the CD36 integrin receptor on monocytes/macrophages^32^, ^33^, ^34^ to amplify macrophage infiltration^52^. Critically, our immunofluorescence co-localization analysis identifies mesangial cells as the primary source of THBS1, with secreted THBS1 binding macrophage CD36. This aligns with established mechanisms wherein THBS1 upregulates macrophage CD36 expression, recruiting immune cells and intensifying inflammation^77^^,^^78^—effects abrogated by THBS1-CD36 blockade *via* inhibitory peptides or antibodies^33^. Our Co-IP data further confirm inflammation-induced THBS1 hypersecretion by mesangial cells. Secreted THBS1 binds to the CD36 receptor on macrophages, activating them and promoting the release of pro-inflammatory cytokines, such as TNF*α*. These cytokines further affect mesangial cells, altering their cell cycle and promoting proliferation. In summary, under inflammatory conditions, mesangial cells secrete THBS1, which binds to the CD36 receptor on macrophages, facilitating their activation and pro-inflammatory factor secretion. CGA treatment effectively reverses this pathological process.

To investigate the upstream molecular mechanism by which CGA exerts anti-inflammatory and anti-proliferative effects *via* modulation of the THBS1, we conducted a streptavidin-biotin affinity pull-down assay. We identified RAC1, a small GTP-binding enzyme, as a direct binding protein of CGA in mesangial cells, the target cells in anti-Thy1 nephritis. RAC1 is an upstream regulator of THBS1^38^, functioning in cytoskeleton remodeling, cell proliferation, and migration^79^. Previous studies have shown that the small GTPase RAC1 is crucial in regulating THBS1 expression^38^. RAC1 activity is primarily controlled by the interconversion of GTP and GDP, resulting in an “on/off” switch mechanism^80^^,^^81^, where RAC1 is active in the presence of GTP and inactive when bound to GDP^82^. In this study, MD analysis and RAC1 activity assessment revealed that CGA suppresses RAC1 activation by forming stable interactions within its GTP/GDP-binding domain. Both CGA and EHT1864 exhibited comparable inhibitory effects on RAC1 activity. When used in combination, these compounds do not result in an additive inhibitory effect on RAC1. The likely mechanism is that CGA competitively binds the same site on RAC1 targeted by EHT1864, thereby inhibiting RAC1 activation. As a natural compound, CGA offers advantages such as accessibility, cost-effectiveness, and minimal side effects. Given its inhibitory impact on RAC1 activity, we propose that CGA could serve as a potential alternative to conventional RAC1 inhibitors, expanding therapeutic options for conditions associated with RAC1 activation.

Further to validate the specific mechanism by which CGA regulates THBS1 expression through RAC1 suppression, we demonstrated that CGA inhibits AKT phosphorylation by suppressing RAC1 activity, thereby reducing THBS1 expression. As a molecular switch, RAC1 regulates AKT activation through multiple mechanisms, interacting with signaling molecules that influence AKT phosphorylation, impacting its effects on cellular functions^62^, ^63^, ^64^. In this study, we confirmed that RAC1 binds to PI3K 110*β*, an interaction that modulates the AKT pathway. Dysregulation of RAC1 and AKT signaling can enhance cell proliferation and survival, contributing to tumorigenesis. Moreover, RAC1's involvement in actin cytoskeleton modulation contributes to the spatial regulation of AKT signaling, a crucial component of efficient cellular responses to growth factors^83^^,^^84^. The transcription factor AKT regulates THBS1 expression *via* both direct and indirect pathways. Notably, AKT directly suppresses THBS1 expression by modulating its promoter activity^85^. Additionally, under conditions of inflammation and hypoxia, AKT can directly influence THBS1 expression^31^^,^^86^. Furthermore, research has shown that AKT exerts an indirect regulatory effect on THBS1 expression by modulating other proteins that influence its levels. For example, AKT2 mediates the balance between procoagulant and anticoagulant factors, thereby indirectly affecting THBS1 levels in the context of venous thrombosis^87^. In contrast, in clear cell renal carcinoma, THBS1 has been shown to regulate AKT phosphorylation, influencing cellular migration and invasion^88^. This observation diverges from the regulatory direction identified in our study, suggesting a potential reciprocal regulatory relationship between AKT and THBS1. Their functional dynamics appear to vary depending on the specific disease state and microenvironmental conditions. Interestingly, even when exogenous THBS1 expression was driven by the strong constitutive CMV promoter—which is generally considered independent of endogenous upstream signaling, such as the RAC1–Akt axis—CGA treatment still reduced total THBS1 protein levels. This observation may imply the potential involvement of post-transcriptional or transcription-independent regulatory mechanisms. For example, CGA could promote THBS1 protein degradation through the ubiquitin–proteasome pathway, possibly by activating specific E3 ubiquitin ligases or inhibiting deubiquitinating enzymes. Alternatively, CGA might accelerate the decay of THBS1 mRNA by modulating RNA-binding proteins, even though the transcript is of exogenous origin. Additionally, given the multi-target nature of CGA, we cannot exclude the possibility that it may indirectly influence CMV promoter activity or plasmid stability within the nucleus. In summary, our study partially corroborates the notion that RAC1 modulates AKT phosphorylation, thereby influencing THBS1 expression, whereas CGA demonstrates anti-inflammatory and anti-proliferative effects by regulating RAC1 activity.

Despite the contributions of this study, several limitations must be acknowledged. Firstly, RAC1 as a target of CGA needs further validation. Although liver and kidney function toxicity assessments were conducted to evaluate safety and rule out significant off-target risks preliminarily, the study design primarily focused on the RAC1 pathway. As a result, the potential synergistic mechanisms between RAC1 and other putative targets of CGA were not thoroughly investigated, which may limit a comprehensive understanding of its overall pharmacological network. Future studies should therefore explore CGA's multi-target interactions in greater depth. Additionally, our research was confined to animal and cellular models, which may not fully replicate the therapeutic effects of CGA in humans. Therefore, before considering CGA for clinical application, it is imperative to establish its efficacy and safety through comprehensive large-scale clinical trials.

## 4 Conclusions

In conclusion, this study investigated the protective effects of CGA on renal function in animal models of MsPGN. Our findings identified RAC1 as a molecular target of CGA, demonstrating that CGA inhibits RAC1 activation by competitive binding to its GTP/GDP-binding domain. This inhibition subsequently suppresses AKT phosphorylation and significantly downregulates THBS1 expression, thereby attenuating the inflammatory response and mesangial proliferation associated with the THBS1‒CD36 interaction. This research first proposes that CGA exerts its protective effects in glomeruli and mesangial cells by targeting the RAC1-AKT-THBS1-CD36 signaling axis, offering new mechanistic evidence for MsPGN treatment. Future studies will explore its protective effects in other renal structures and cell types, potentially expanding its therapeutic applications.

## 5 Experimental

### 5.1 Cell culture and treatments

CGA (C_16_H_18_O_9_; molecular weight: 354.31) was procured from MedChemExpress. Primary HRMCs (4200, ScienCell) were seeded into a 6-well plate and cultured in cell medium (4201, ScienCell) supplemented with 2% fetal bovine serum (0010, ScienCell), 1% penicillin/streptomycin solution (0503, ScienCell), and 1% cell growth factor (4252, ScienCell) in humidified air with 5% CO_2_ at 37 °C. TNF*α* (40 ng/mL, 300-01A, Peprotech), ML-097 (HY-118208, MCE), EHT1864 (HY-16659, MCE), AKT inhibitor Ⅷ (HY-10355, MCE), and SC79 (HY-18749, MCE) were used as stimulants for HRMCs.

The plasmid harboring the cytomegalovirus (CMV) promoter to drive THBS1 expression, as well as its corresponding control vector, were purchased from GeneCopoeia.

### 5.2 Animal treatment

Male Wistar rats, weighing 190–220 g and aged 6 weeks, were procured from SPF (Beijing, China) Biotechnology Co. After a 1-week acclimatization period, the rats were randomly assigned to one of five groups (six rats per group): (i) control group (Wistar rats treated with normal saline); (ii) Day 3 model group (Wistar rats treated with anti-Thy1 antibodies); (iii) Day 3 model + CGA group (Wistar rats treated with anti-Thy1 antibodies and CGA); (iv) Day 7 model group (Wistar rats treated with anti-Thy1 antibodies); (v) Day 7 model + CGA group (Wistar rats treated with anti-Thy1 antibodies and CGA). To study CGA’s impact on anti-Thy1 nephritis, rats were administered anti-Thy1 antibodies (2.5 mg/kg) intravenously to induce the condition; the control group received saline. Rats in the CGA treatment groups were administered intraperitoneal injections of CGA solution (10 mg/kg) daily for 7 consecutive days; the control and model groups received an equivalent volume of cosolvent. The dosage was based on a comprehensive review of the literature and preliminary trials. On Days 3 and 7, rats were euthanized, and kidney tissues, blood, and urine samples were collected.

The experimental protocol for BAFF mice is described in Supporting Methods S1.

The experiments adhered to the ARRIVE guidelines and the EU Directive 2010/63/EU on animal experiments, and were approved by the Animal Ethics Committee of the Chinese PLA General Hospital (2020-X16-90).

### 5.3 Assessment of renal pathology

Kidney specimens were fixed in 10% formalin, embedded in paraffin, and sectioned at 4 μm thickness, followed by Periodic Acid-Schiff (PAS) staining to evaluate glomerular cell proliferation. Twenty glomeruli per section were selected for analysis.

### 5.4 Quantitative real-time polymerase chain reaction (qRT-PCR)

The Trizol method (Thermo Fisher Scientific, Waltham, MA, USA) was utilized to extract total RNA from cells and kidney tissue according to the manufacturer’s instructions. We then reverse-transcribed 1 g of total RNA using the ProtoScript II First-Strand cDNA Synthesis Kit (E6560S, New England Biolabs, Frankfurt, Germany). The qRT-PCR analysis utilized PowerUp SYBR Green Master Mix (Applied Biosystems) and primers listed in Supporting Information Table S1. The QuantStudioTM 5 Real-Time System (ThermoFisher Scientific, CA, USA) was used for qRT-PCR analysis. Data normalization was based on the expression levels of the 18S and GAPDH genes, and relative gene expression was determined using the comparative threshold cycle (Delta Ct) method.

### 5.5 Western blotting

Renal tissues or cells were lysed in RIPA buffer with added protease inhibitors, including 1 μg/mL leupeptin, 1 μg/mL aprotinin, and 100 μmol/L PMSF. Following a 30-min incubation, the lysates were centrifuged at 13,000×*g* and 4 °C for 30 min. Protein concentrations were measured using a BCA protein assay kit (Thermo Fisher Scientific, Waltham, MA, USA). Each sample, containing approximately 30 μg of protein, was subjected to either 8% or 10% SDS-PAGE, followed by transfer to a membrane. The membranes were blocked and incubated with primary antibodies: Anti-Thrombospondin 1 (1:1000, ab267388, Abcam; 1:1000, 37879S, CST), *p*-AKT (1:1000, 13038S, CST), AKT (1:1000, 9272S, CST), CD36 (1:1000, ABclonal, A5792), Cyclin E1 (1:1000, ab7959, Abcam), Cyclin D1 (1:1000, 60186-1-lg, Proteintech), *β*-Actin (1:20000, 66009-1-Ig, Proteintech), and Vinculin (1:10000, 66305-1, Proteintech) at 4 °C for 20 h. Secondary antibody incubation occurred at 4 °C for 3 h. WB analysis was conducted using ImageJ software version “ImageJ 154f” (National Institutes of Health, Bethesda, MD, USA). Each experiment was repeated at least three times.

### 5.6 Statistical analysis

Data were analyzed using GraphPad Prism 10 software (GraphPad Software, San Diego, CA) and are presented as the mean ± standard error of the mean (SEM). For data collected at a single time point, a one-way analysis of variance (ANOVA) was used for groups with 3 or more samples, followed by a *post hoc* multiple-comparison test. For data collected at various time points, a two-way ANOVA was used, followed by a post hoc multiple-comparison test. Statistical significance was defined as a *P*-value <0.05 for all comparisons.

### 5.7 Data statement

The mass spectrometry proteomics data have been deposited in the ProteomeXchange Consortium (https://proteomecentral.proteomexchange.org) *via* the iProX partner repository with the dataset identifier PXD058367.

## Author contributions

Xiangmei Chen, Quan Hong, Ping Li, Guangyan Cai and Lingling Wu, Yong Wang: conceptualization and methodology. Yilun Qu, Quan Hong: Original draft preparation, writing, review, and editing. Yilun Qu, Fei Peng, Jie Zhang, Dan Pan, Tiantian Wang, Yena Zhou, Jikai Xia, Jiayi He, Chunru Shi, Yinghua Zhao, Ran Liu, Yingjie Zhang, Jiaona Liu: Investigation. Lingling Wu, Jie Wu, Shuwei Duan: Validation. Yilun Qu, Xu Wang: Formal analysis. Xiangmei Chen, Quan Hong, Ping Li, and Guangyan Cai: Supervision.

## Conflicts of interest

The authors have no conflicts of interest to disclose.

## Appendix A Supporting information

The following is the Supporting information to this article.
