# Supplementary material for: Direct pharmacological targeting of RAC1 by chlorogenic acid: A novel therapeutic approach for mesangial proliferative glomerulonephritis
Source: Acta Pharm Sin B. 2026 Jun 24;16(9):6080–98. doi: 10.1016/j.apsb.2026.06.041 (PMC13589924; doi:10.1016/j.apsb.2026.06.041)
Supplement: Supplementary file 1 [file mmc1.pdf]

Supporting Information for

Original article

Direct pharmacological targeting of RAC1 by chlorogenic acid: A novel therapeutic approach for mesangial proliferative glomerulonephritis

Yilun Qu[a,†], Lingling Wu[a,†], Yong Wang[a,†], Fei Peng[a,b], Jie Zhang[a,b], Dan Pan[a,c], Tiantian Wang[a], Yena Zhou[a,b], Jikai Xia[a,c], Jiayi He[d], Chunru Shi[a,c], Yinghua Zhao[e], Xu Wang[a], Ran Liu[a], Yingjie Zhang[a], Jiaona Liu[a], Jie Wu[a], Shuwei Duan[a], Guangyan Cai[a,*], Ping Li[a,*], Quan Hong[a,*], Xiangmei Chen[a,*]

[a]*Senior Department of Nephrology, Chinese PLA General Hospital, State Key Laboratory of Kidney Diseases, National Clinical Research Center for Kidney Diseases, Beijing Key Laboratory of Medical Devices and Integrated Traditional Chinese and Western Drug Development for Severe Kidney Diseases, Beijing Key Laboratory of Digital Intelligent TCM for the Prevention and Treatment of Pan-vascular Diseases, Key Disciplines of National Administration of Traditional Chinese Medicine, Innovation Team and Talents Cultivation Program of National Administration of Traditional Chinese Medicine, Beijing 100853, China*

[b]*School of Medicine, Nankai University, Tianjin 300071, China*

[c]*Institute of Chinese Medicine, Guangdong Pharmaceutical University, Guangzhou 510006, China*

[d]*Clinical Research Center, The First Affiliated Hospital of Shantou University Medical College, Shantou 515041, China*

[e]*Beijing Tsinghua Changgung Hospital, School of Clinical Medicine, Tsinghua University, Beijing 102218, China*

Received 13 October 2025; received in revised form 21 January 2026; accepted 19 May 2026

[†]These authors made equal contributions to this work.

[*]Corresponding authors.

E-mail addresses: xmchen301@126.com (Xiangmei Chen), hongquan@301hospital.com.cn (Quan Hong), liping.8@163.com (Ping Li), caiguangyan@sina.com (Guangyan Cai).

Running title: CGA targets at RAC1 treating MsPGN

This file includes：

Supplementary Methods:

1 Experimental Protocol for BAFF Mice
2 Analysis of urine samples
3 Immunohistochemical and immunofluorescence staining
4 Laser Micro-Cutting Proteomics
5 Pull-down assay
6 Computer modeling and docking
7 Molecular dynamics simulations
8 Co-culture of HRMCs and macrophages
9 Detection and quantification of THBS1 secretion in HRMCs
10 Co-immunoprecipitation
11 Surface Plasmon Resonance (SPR) Analysis
12 RAC1 activity assay
13 CCK-8 assay
14 EDU assay

Figure S1 Chlorogenic Acid (CGA) dose optimization and safety evaluation in anti-Thy1 nephritis

Figure S2 Proteome GO analysis, representative DEP mRNA expression, and WB stats of glomerular AKT phosphorylation and THBS1 fluorescence in rats

Figure S3 Improvement of glomerular inflammation and proliferation in CGA-treated BAFF mice and amelioration of glomerular fibrosis in anti-Thy1 nephritis rats on day 7

Figure S4 Mass Spectrometry (MS) and Nuclear Magnetic Resonance (NMR) for Bio-CGA, along with Protein-Protein Docking Analysis and the Tandem Mass Spectrometry (MS/MS) Graph of RAC1

Figure S5 Dynamic cross-correlation matrix (DCCM) of the compound-RAC1 protein complex, and SPR detection of point-mutated RAC1 protein with CGA

Figure S6 CGA cytotoxicity assessment, cell experimental dose screening, statistical analysis of *α*-SMA immunofluorescence expression, as well as THBS1 Immunofluorescence in HRMCs after Treatment with Different Doses of CGA

Figure S7 Co-IP assay for the binding ability between RAC1 and PI3K 110β, and schematic of the cell co-culture system

Table S1 Summary of primer sequences

Table S2 Mass spectrometer search parameters

1. *Experimental Protocol for BAFF Mice*

Male BAFF-Tg and WT mice, weighing 18-22 g, were obtained from GemPharmatech Co. Ltd. (Beijing, China). The animals were housed in a controlled environment with standard conditions and provided ad libitum access to food and water. Starting at 12 weeks of age, mice in the CGA treatment groups were administered intraperitoneal injections of CGA solution (20 mg/kg) daily for 4 weeks, consecutive days; the control and model groups received an equivalent volume of cosolvent. At 16 weeks of age, the mice were euthanized, and kidney tissues, blood, and urine samples were collected for further analysis.

2. *Analysis of urine samples*

Urine samples were collected at random intervals to determine the UACR. The collected urine was centrifuged at 800 × g for 30 min, followed by separation of the supernatant into 1.5-mL tubes and storage at -80°C until later analysis. The concentration of urinary creatinine was quantified using a creatinine assay kit from Cayman Chemical (500701). At the same time, urine albumin levels were assessed using a commercially available ELISA kit (E11-125) from Bethyl Laboratory Inc.

3. *Immunohistochemical and immunofluorescence staining*

Immunohistochemical staining was performed according to established protocols. Specifically, the kidney sections were dewaxed, treated with 3% H_2O_2, and heated to facilitate antigen retrieval. Subsequently, the sections were incubated overnight at 4 °C with primary antibodies, including anti-CD68 (1:200, ab125212, Abcam) and α-SMA (1:200, ab7817, Abcam). Following rinsing with phosphate-buffered saline (PBS), the sections were incubated with a secondary antibody and then exposed to diaminobenzidine (DAB kit, Vector Laboratories).

Renal tissue sections were deparaffinized in xylene and rehydrated, then permeabilized with 0.1% Triton for 10 min. Subsequently, the sections were left to incubate overnight at 4 °C with primary antibodies targeting Desmin (1:200, SANTA), PCNA (1:500, ab92552, Abcam), Ki67 (1:500, TW0001S, Abmart), Cyclin E1(1:200, ab7959, Abcam), CDK4 (1:200, ab7955, Abcam), anti-Thy1 (1:800), and THBS1 (1:100, ab267388, Abcam). Subsequently, the sections underwent three washes in PBS and were incubated with secondary antibodies. Following this, staining with a fluorophore was performed for 1 h at ambient temperature. After another round of washing, the sections were mounted onto glass slides with Fluoroshield Mounting Medium, and imaging was performed using confocal fluorescence microscopy.

Cells were grown in 24-well dishes, exposed to TNFα, fixed with 4% paraformaldehyde for 30 min, and subsequently blocked with goat serum at room temperature for 1 h. Following PBS washing, primary antibodies α-SMA (1:500, ab7817, Abcam) and THBS1 (1:100, ab267388, Abcam) were applied, and the cells were incubated at 4°C overnight. Then, the cells were washed 3 times with PBS and incubated with the Alexa Fluor fluorophore for 2 h at room temperature. Following staining of the nuclei with DAPI, the cells were observed using a confocal microscope.

4. *Laser Micro-Cutting Proteomics*

The tissue excision was performed using a 337-nm nitrogen laser under an inverted microscope. A single beam at a specified laser intensity was utilized to remove the cutting portion for sample collection. Each group included four samples, yielding a total of 156 glomeruli per individual.

The label-free technique in quantitative proteomics relies on the relative signal intensities of mass spectrometry peaks to quantify peptides or proteins, rather than using isotope markers for peptide labeling. Differential proteins were identified using a standard threshold of ratio> 1.5 or ratio< -1.5, along with a significance level of $p < 0.05$. Volcano plot, Venn diagram, Heatmap, and GO and KEGG pathway enrichment analyses were performed using https://www.bioinformatics.com.cn (last accessed on 10 Nov 2023), an online platform for data analysis and visualization.

5. Pull-down assay

Biotin labeling was performed to introduce a biotinylated CGA with a PEG chain, followed by assessment of CGA activity post-labeling. Cell lysis for protein extraction used a specialized WB and IP lysis solution for mesangial cell lysis, supplemented with a 1× protease inhibitor cocktail. In the pulldown experiment, the fixed streptavidin gel was equilibrated, biotin-labeled small molecules and biotin were immobilized, biotin blocking was conducted, biotin-labeled small molecules were captured, and a Clean Spin Column tube was used. Before protein enzymatic digestion, protein samples were pretreated with reduction and alkylation, followed by enzymatic digestion, peptide extraction, and loading onto beads. Proteome Discoverer 2.4 software was used to retrieve and analyze the data files, using the search library parameters outlined in Table S2.

The chemical characterization information for the biotinylated chlorogenic acid (Bio-CGA) probe is as follows: ① Mass Spectrometry (MS): High-resolution mass spectrometry (HRMS) data confirmed the successful synthesis of Bio-CGA. The theoretical m/z value of the target molecule is 783.3481, and a distinct peak was observed at m/z = 783.3488 in the mass spectrum. The relative error is calculated as follows: (783.3488 - 783.3481)/783.3481 × 100% = 0.000089% (i.e., 0.89 ppm), which is less than 5 ppm. ②Nuclear Magnetic Resonance (NMR): The ^{1}H NMR spectrum was provided, and the assignment of all peaks has been completed. As shown in the ^{1}H NMR spectrum, the baseline is flat with no prominent impurity peaks, which is consistent with the structure of the target compound. The MS and NMR spectra are shown in Figure S4A, B.

6. Computer modeling and docking

The 3D structure of CGA was sourced from the PubChem database and refined with Schrodinger's LigPrep module utilizing the OPLS_4 force field. The Epik module was then utilized to identify stereoisomers and protonated states.

Protein preparation and docking: (i) The RAC1 protein, obtained from the Uniprot database with human species designation, was chosen for analysis due to its high-resolution crystal structure (PDB ID: 1MH1) and its interaction with small molecule compounds. The Protein Preparation Wizard module in Schrodinger software streamlined the protein preparation process by executing essential operations, including bond assignment, hydrogenation, completion of missing side chains, removal of water molecules, and cofactors within the protein. This was subsequently followed by the enhancement of the hydrogen-bonding network and energy reduction using the OPLS_4 force field. (ii) The docking process involved the utilization of the Glide module within the Schrodinger software to conduct molecular docking. The original crystallized small-molecule ligand was used as a binding pocket, with outer box dimensions set to match those of the original small-molecule compound and an inner box size set to a distance range of 10 Å. Docking simulations were carried out in standard precision (SP-docking) mode, employing the Glide Score as the scoring function provided by Schrodinger. For each small molecule compound, 10 conformations were generated during the docking simulation. These conformations underwent post-docking analysis and minimization to identify the conformation with the highest score for subsequent analysis purposes.

7. Molecular dynamics simulations

Molecular dynamics simulations were performed using GROMACS (version 2021.6) to investigate interactions between chlorogenic acid (CGA), the reference ligand EHT1864, and the target protein RAC1. Ligand geometries were optimized at the DFT level using ORCA with the r2SCAN-3c functional, followed by single-point energy calculations at the B3LYP-GD3/def2-TZVP level employing def2/J auxiliary basis sets and RIJCOSX approximation. GAFF2 force field parameters generated via Antechamber were assigned to ligands, with RESP2 charges fitted using Multiwfn,

while RAC1 topology was derived from the Amber14SB force field. All systems were solvated in cubic TIP3P water boxes, with counterions to neutralize charge. Energy minimization involved steepest descent (convergence threshold: 1,000 kJ/mol·nm) followed by conjugate gradient (100 kJ/mol·nm). Systems were equilibrated under NVT (1 ns, 298.15 K, V-rescale thermostat) and NPT (1 ns, 1 bar, Parrinello-Rahman barostat) ensembles. Production simulations (100 ns, 298.15 K) used LINCS constraints for hydrogen bonds and the Particle-Mesh Ewald method for electrostatics (1.0 nm cutoff).

8. Co-culture of HRMCs and macrophages

Tohoku Hospital Pediatrics-1 (THP-1) cells (TIB-202, ATCC) were cultured in the upper chamber of a co-culture dish and stimulated with PMA for 24 hours to induce their transformation into M0 macrophages. Subsequently, the medium was changed to regular RPMI 1640 for continued culture. For the cell migration experiment, Transwell culture plates (CLS3422, Corning) were used. THP-1 cells were cultured in the upper chamber of a co-culture dish and stimulated with PMA for 24 hours to induce their transformation into M0 macrophages. Subsequently, the medium was changed to regular RPMI 1640 for co-culture. HRMCs were cultured in the lower chamber. After 24 h of co-culture, the medium in the upper chamber was aspirated, and the chamber was washed twice with PBS. The insert was immersed in a 4% paraformaldehyde solution for 20 min and air-dried. It was then stained with a 0.3% crystal violet solution for 20 min. Non-migrated cells on the insert's top surface were gently removed with a cotton swab, followed by three rinses with PBS. Ten random fields were observed under a microscope to count and analyze cells.

For the co-immunoprecipitation (Co-IP) experiment and detection of macrophage inflammatory factors, a Transwell co-culture dish (CLS3450, Corning) was used. HRMCs were cultured in the upper chamber, while THP-1 cells, stimulated with PMA for 24 h to become macrophages and subsequently switched to standard 1640 complete medium, were cultured in the lower chamber. After 24 h of co-culture, the upper chamber was discarded, and the medium in the lower chamber was aspirated. Cells were then trypsinized to extract protein and RNA for subsequent analysis.

9. Detection and quantification of THBS1 secretion in HRMCs

Detection was performed using the Human THBS ELISA Kit (JL11889, Jianglai Biosciences) following a specific protocol: i) centrifuge HRMC supernatant at 1000×*g* for 20 min at 4°C and dilute the supernatant by a factor of universal diluent; ii) prepare standard solutions as per instructions; iii) add 100 μL of samples or standard solutions to respective wells. Add 100 μL of universal diluent to blank wells. Set up duplicates for each condition and incubate at 37°C with sealing film for 60 min; iv) remove the liquid and introduce 100 μL of biotinylated antibody working solution into each well. Cover and incubate at 37℃ for 60 minutes. v) discard the liquid and wash the plate three times; vi) dispense 100 μL of enzyme conjugate working solution into each well, cover, and incubate at 37°C for 30 minutes. vii) discard the liquid and wash the plate vigorously three times; viii) Add 90 μL of TMB substrate and incubate at 37°C in the dark for 15 minutes. Then, add 50 μL of stop solution to each well and promptly measure the optical density at 450 nm.

10. Co-immunoprecipitation

The Co-IP was performed using the Immunoprecipitation kit (Proteintech, PK10007) as follows: i) collect macrophages from the co-culture system and resuspend them in IP lysis buffer; ii) sonicate the cell lysate at 180 W power for 1 min; iii) place on ice for 30 minutes, inverting every 10 min; iv) centrifuge at 10,000×g for 10–15 min at 4°C and transfer the supernatant to new EP tubes for IP and WB experiments; v) determine lysate protein concentration using a BCA assay; vi) wash rProtein A/G beads slurry with low-speed centrifugation in PBS three times and resuspend to the

original volume in PBS; vii) introduce resuspended rProtein A/G bead slurry into the EP tubes containing lysate; viii) incubate with rotation at 4°C for 30-60 min; ix) centrifuge at 500×*g* for 1 minute at 4°C and transfer the supernatant to new EP tubes; x) combine 300 µL of lysate (1 mg total protein) with specific CD36 antibody, IgG antibody, and incubation buffer in capped spin columns. Incubate overnight at 4°C; xi) add prepared rProtein A/G beads slurry to spin columns to precipitate immune complexes and incubate with rotation at 4°C for 3 h; xii) remove end caps and discard naturally flowing supernatant; xiii) wash precipitated complexes five times, centrifuging for 30 seconds each time and discarding the supernatant; xiv) collect the eluted product, add 10 µL of alkali neutralization buffer and 23 µL of 5× sample buffer, and heat in a boiling water bath for 5 min; xv) perform WB detection.

11. Surface Plasmon Resonance (SPR) Analysis

The CM5 sensor chip was activated with freshly mixed 400 mM EDC/100 mM NHS at 10 µL/min for 420 s, followed by immobilization of RAC1 protein (20 µg/mL in immobilization buffer) on flow cell Fc2 achieving ≈12,600 RU response, while Fc1 served as a reference flow cell; unbound sites were blocked with 1 M ethanolamine-HCl (pH 8.5, 10 µL/min, 420 s). Multi-cycle kinetics were performed by injecting serially diluted CGA (1–100 µM) sequentially: association phase (20 µL/min, 100 s), dissociation phase (analyte buffer, 180 s), with surface regeneration using 10 mM glycine-HCl (pH 2.0, 30 µL/min, 30 s) between cycles.

12. RAC1 activity assay

The Rac1 G-LISA Activation Assay Kit (Cytoskeleton BK128) was used according to the manufacturer's instructions. Duplicate wells received 50 µL each of cell lysate and RAC1 Control Protein, then were shaken on an orbital microplate shaker at 200 rpm and 4°C for 30 min. Next, 200 µL of Antigen Presenting Buffer at room temperature was added to each well, and the mixture was incubated for 2 min. Subsequently, 50 µL of diluted anti-Rac1 primary antibody was added to each well, and the plate was shaken at 200 rpm at room temperature for 45 min. This was followed by the addition of 50 µL of diluted Secondary antibody to each well, and the plate was again placed on the shaker at room temperature for another 45 min. Then, 50 µL of Mixed HRP detection reagent was added to each well and incubated for 20 minutes at room temperature. Finally, 50 µL of HRP Stop Buffer was added to each well, and the absorbance was measured at 450 nm using a microplate spectrophotometer to determine the signal. Wells that contained only Lysis Buffer served as the assay's Blank.

13. CCK-8 assay

Cell viability was assessed using a Cell Counting Kit-8 (CK04, Dojindo). HRMCs were seeded in 96-well plates and exposed to varying concentrations of CGA. Subsequently, the absorbance at 450 nm was determined following a 2.5-h incubation with 10 µL of CCK-8 reagent per well.

14. EDU assay

Cell proliferation was assessed following the manufacturer's instructions, using the BeyoClick™ Equal Concentration 20 µmol/L 5-ethynyl-2'-deoxyuridine (EDU) Cell Proliferation Kit with Alexa Fluor 555 Imaging Kit (C0075S, Beyotime). HRMCs were cultured in 24-well plates with an EDU added to the medium. The cells were then incubated for 4.8 h. After fixation, HRMCs were incubated with the Click reaction solution in the dark for 30 min at ambient temperature, followed by nuclear staining with Hoechst 33342. The cells were analyzed under a fluorescence microscope from Olympus in Tokyo, Japan. Each experiment was conducted at least three times.

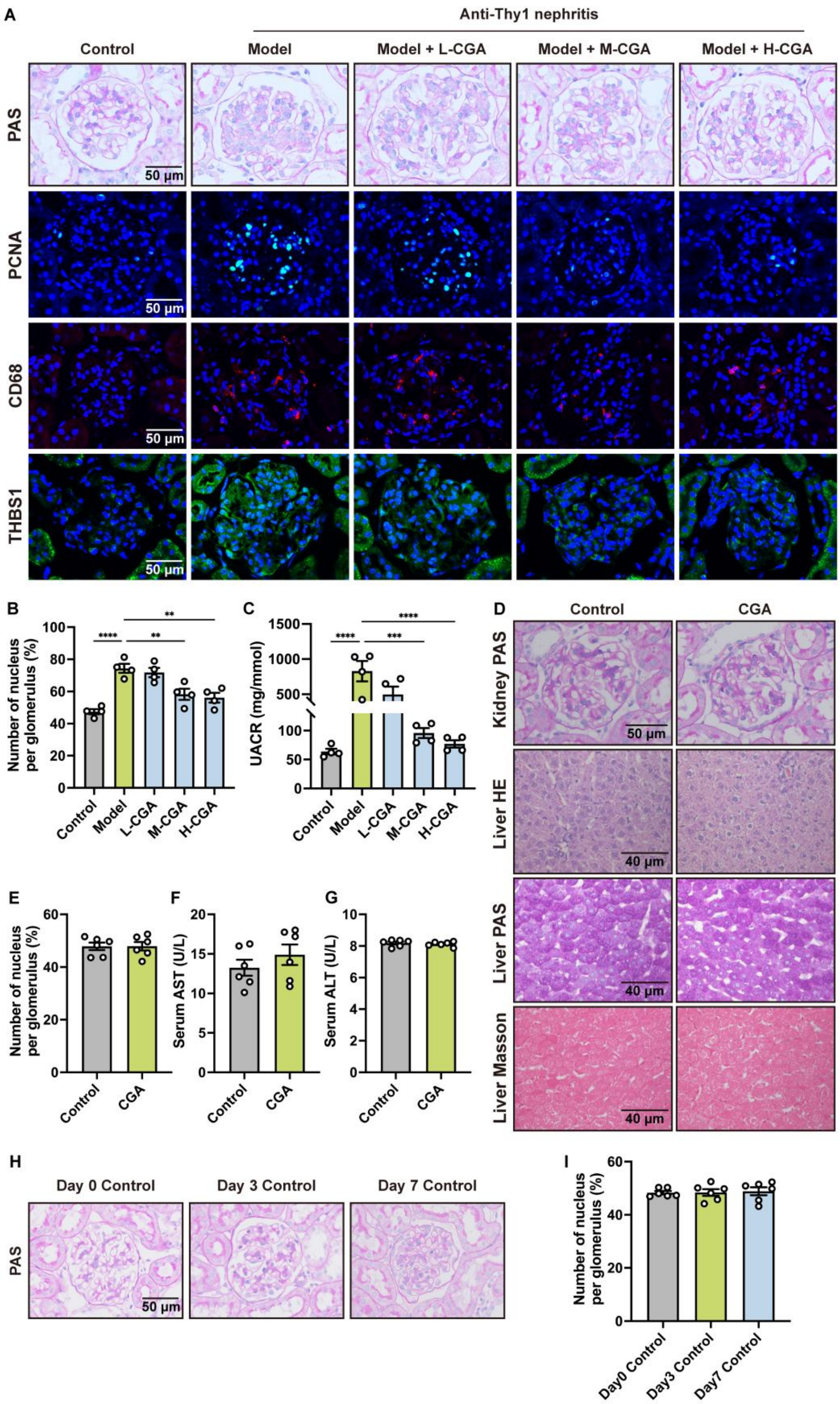

Figure S1 Chlorogenic Acid (CGA) dose optimization and safety evaluation in anti-Thy1 nephritis. (A) Representative images of PAS, PCNA (blue fluorescence indicates DAPI, green fluorescence indicates PCNA), CD68 (blue fluorescence indicates DAPI, red fluorescence indicates CD68), and THBS1 (blue fluorescence indicates DAPI, green fluorescence indicates

THBS1) (Scale bar = 50 μm). (B) Quantification of the number of nuclei per glomerulus after drug treatment (mean ± SEM, n = 4, one-way ANOVA, $^{**}P < 0.01$, $^{****}P < 0.0001$). (C) Urinary albumin-to-creatinine ratios (UACR) of spot urine collected during the study are shown (mean ± SEM, n = 4, one-way ANOVA, $^{***}P < 0.001$, $^{****}P < 0.0001$). (D) Representative renal PAS staining images, and hepatic HE, PAS, and Masson's trichrome staining images for CGA toxicity assessment (Scale bar = 50 μm and 40μm). (E) Quantification of the number of nuclei per glomerulus after drug treatment (mean ± SEM, n = 6, one-way ANOVA, P > 0.05). Statistical Results of Liver Function Test Indicators (F) AST and (F) ALT (mean ± SEM, n = 6, one-way ANOVA, P > 0.05). (H, I) PAS staining of glomeruli and nucleated cell count analysis in control rats across different time points (Scale bar = 50 μm, mean ± SEM, n = 6, one-way ANOVA, P > 0.05).

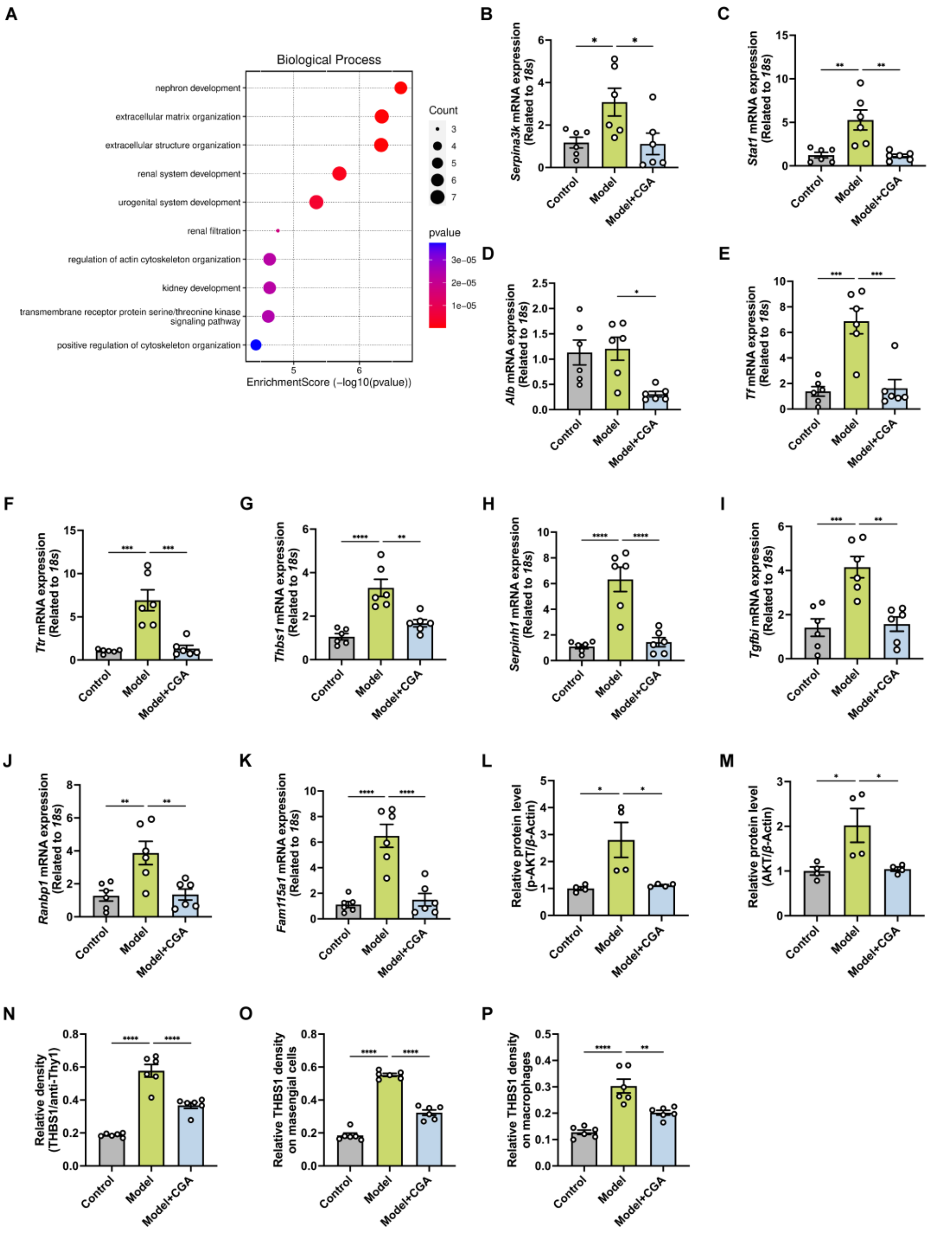

Figure S2 Proteome GO analysis, representative DEP mRNA expression, and WB stats of glomerular AKT phosphorylation and THBS1 fluorescence in rats. (A) Laser microdissection proteomics GO protein functional analysis. (B-K) Statistical analysis of representative differential protein mRNA levels in proteomics (mean ± SEM, n = 6, one-way ANOVA, *P < 0.05, **P < 0.01, ***P < 0.001, ****P < 0.0001). (L-M) Statistical analysis of glomerular p-AKT and AKT protein levels in anti-Thy1 nephritis rats (mean ± SEM, n = 6, one-way ANOVA, *P < 0.05). (N) Statistical analysis of glomerular mesangial cell THBS1 expression via immunofluorescence (mean ± SEM, n = 6, one-way ANOVA, ****P < 0.0001). (O) Statistical analysis of THBS1 and mesangial cell marker anti-Thy1 fluorescence colocalization (mean ± SEM, n = 6, one-way ANOVA, ****P < 0.0001. (P) Statistical analysis of THBS1 and macrophage marker CD68 fluorescence colocalization (mean ± SEM, n = 6, one-way ANOVA, **P < 0.001 ****P < 0.0001).

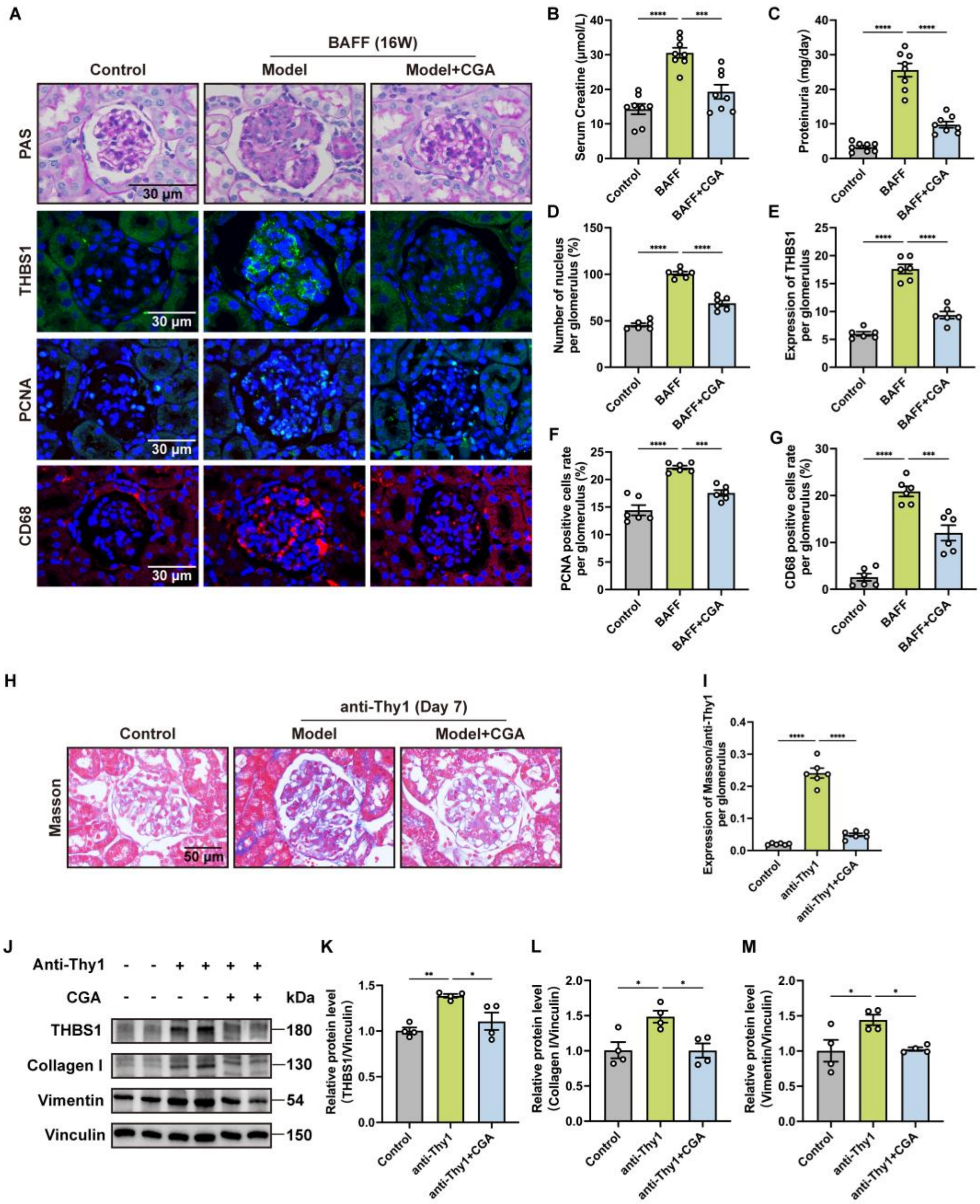

Figure S3 Improvement of glomerular inflammation and proliferation in CGA-treated

BAFF mice and amelioration of glomerular fibrosis in anti-Thy1 nephritis rats on day 7. (A) Representative images of PAS, THBS1 (blue fluorescence indicates DAPI, green fluorescence indicates THBS1), PCNA (blue fluorescence indicates DAPI, green fluorescence indicates PCNA), and CD68 (blue fluorescence indicates DAPI, red fluorescence indicates CD68) (Scale bar = 30 μm). (B) Serum creatinine and (C) proteinuria were measured (mean ± SEM, n = 8, two-way ANOVA, $P > 0.05$). Quantification of (D) PAS, (E) THBS1, (F) PCNA, and (G) CD68 expression after drug treatment (mean ± SEM, n = 6, one-way ANOVA, $^{***}P < 0.001$, $^{****}P < 0.0001$). (H, I) Representative Masson's trichrome-stained glomeruli at day 7 in the anti-Thy1 nephritis model with collagen deposition analysis (Scale bar = 50 μm, mean ± SEM, n = 6, one-way ANOVA, $^{****}P < 0.0001$). (J-M) Western blot analysis of THBS1, Collagen I, and Vimentin protein expression in glomeruli at day 7 post-anti-Thy1 induction, with densitometry normalized to Vinculin (mean ± SEM, n = 4, one-way ANOVA, $^{*}P < 0.05$, $^{**}P < 0.01$).

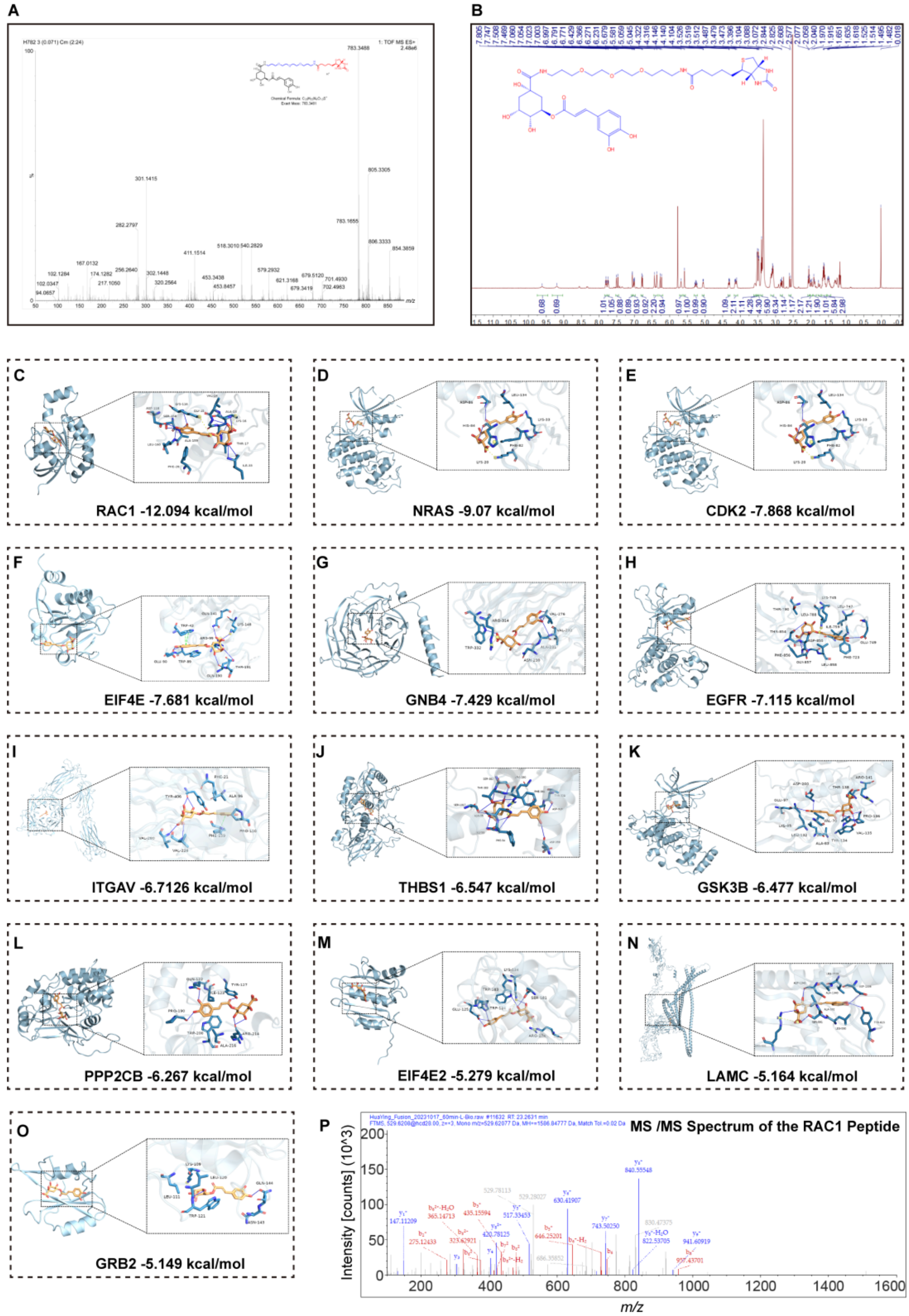

Figure S4 Mass Spectrometry (MS) and Nuclear Magnetic Resonance (NMR) for Bio-CGA, along with Protein-Protein Docking Analysis and the Tandem Mass Spectrometry (MS/MS) Graph of RAC1. (A) MS for Bio-CGA. (B)NMR for Bio-CGA. (C-O) Protein-protein docking analysis reveals direct interactions between CGA and HRMCs cleavage products on the PI3K-AKT pathway. (P) Profile of the RAC1 peptide.

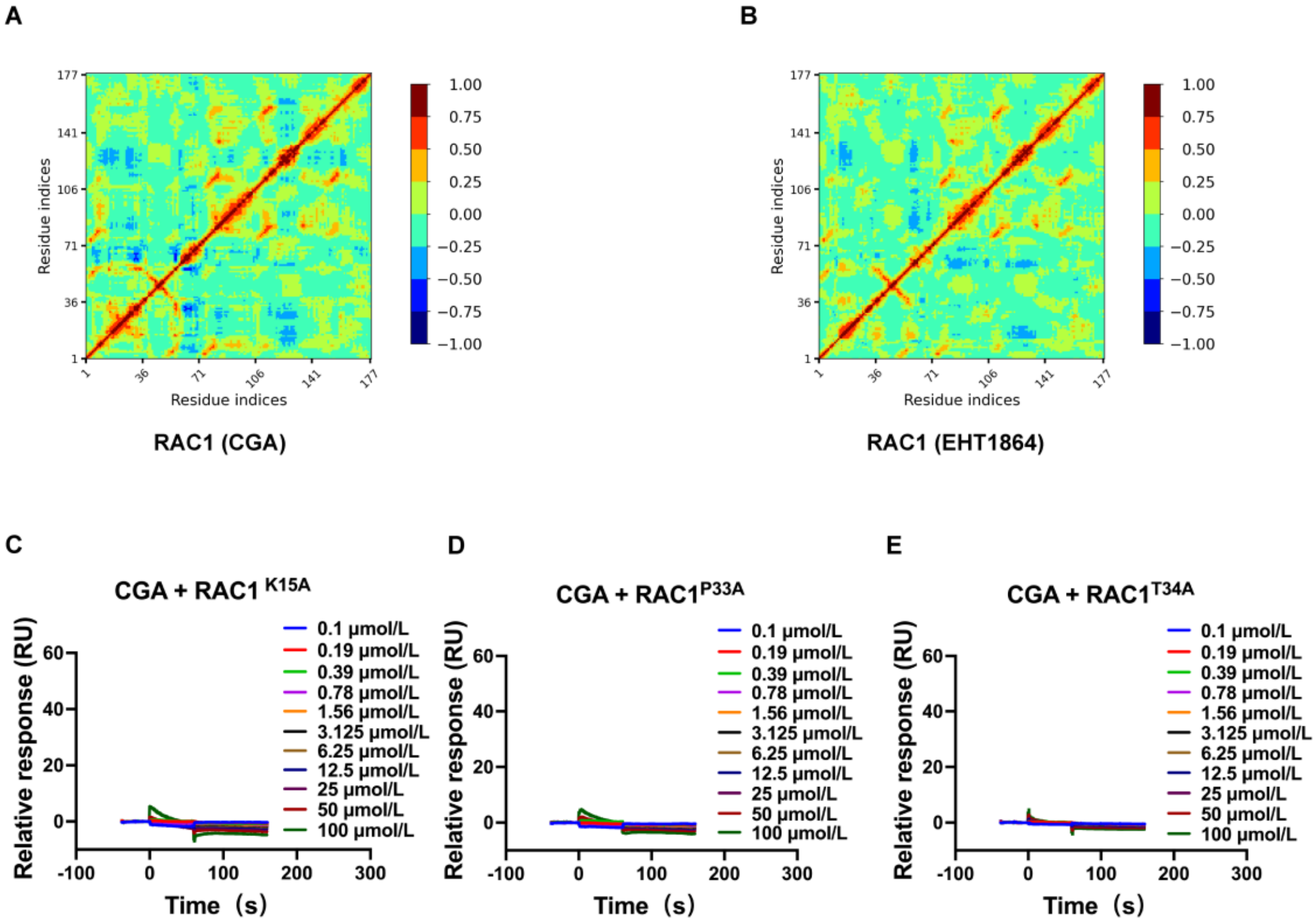

Figure S5 Dynamic cross-correlation matrix (DCCM) of the compound-RAC1 protein complex, and SPR detection of point-mutated RAC1 protein with CGA. (A) Dynamic cross-correlation matrix (DCCM) of CGA-RAC1 complex. (B) Dynamic cross-correlation matrix (DCCM) of EHT1864-RAC1 complex. (C) SPR detection of LYS15 site-mutated RAC1 protein with CGA. (D) SPR detection of PRO33 site-mutated RAC1 protein with CGA. (E) SPR detection of THR34 site-mutated RAC1 protein with CGA.

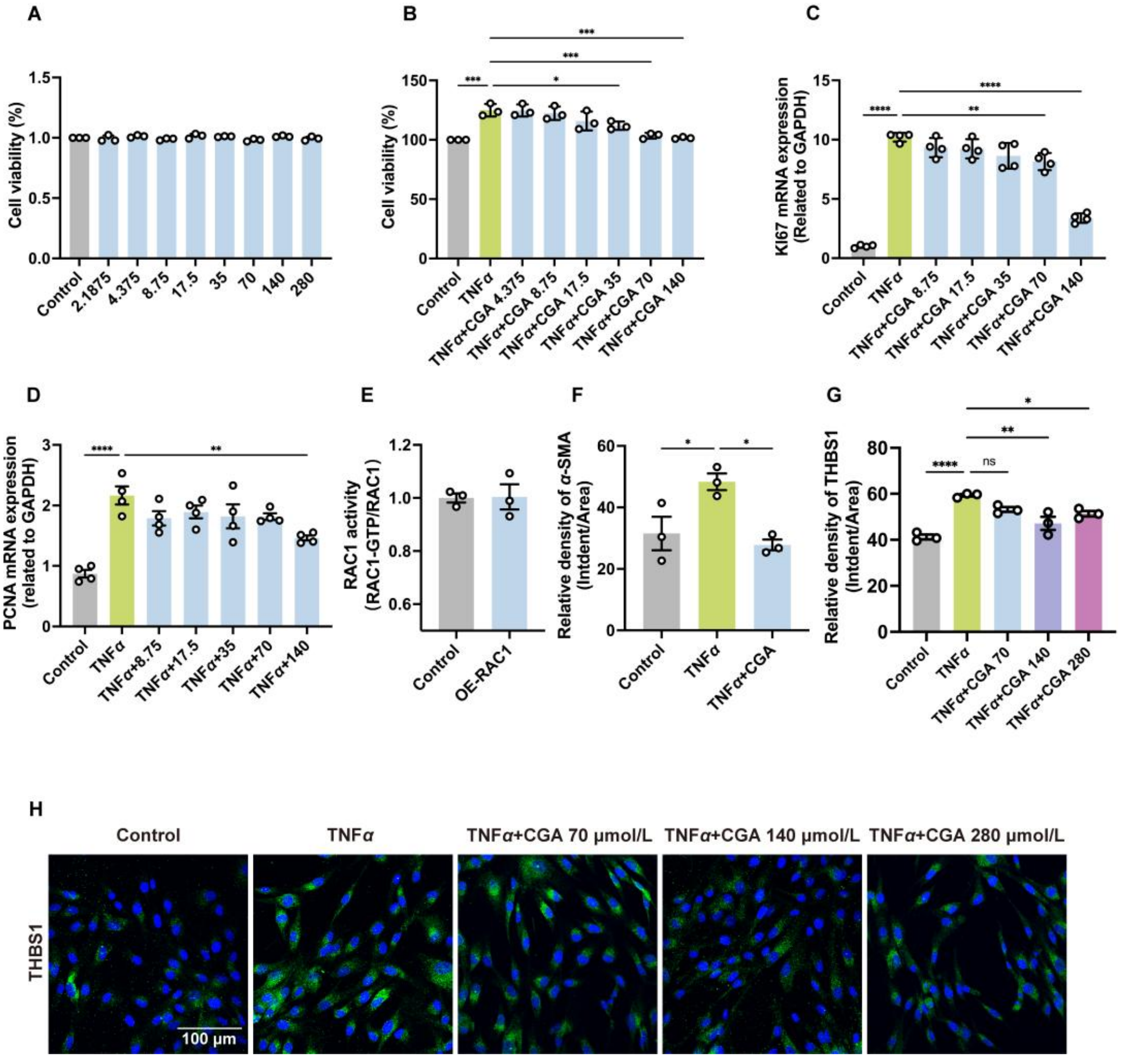

Figure S6 CGA cytotoxicity assessment, cell experimental dose screening, statistical analysis of *α*-SMA immunofluorescence expression, as well as THBS1 Immunofluorescence in HRMCs after Treatment with Different Doses of CGA. (A) After cell adhesion, cells were treated with different concentrations of CGA for 48 h, followed by CCK-8 assay to detect cell viability (mean ± SEM, n = 3, one-way ANOVA, $P > 0.05$). (B) Mesangial cells were seeded and stimulated with 40 ng/mL TNF*α* to establish a proliferation model, then treated with different concentrations of CGA for 48 h, and a CCK-8 assay was used to detect cell proliferation (mean ± SEM, n = 3, one-way ANOVA, $^{*}P < 0.05$, $^{***}P < 0.001$). (C) Detection of Ki67 mRNA expression levels as a proliferation marker (mean ± SEM, n = 4, one-way ANOVA, $^{**}P < 0.01$, $^{****}P < 0.0001$). (D) Detection of PCNA mRNA expression levels as a proliferation marker(mean ± SEM, n = 4, one-way ANOVA, $^{**}P < 0.01$, $^{****}P < 0.0001$). (E) Assay of RAC1 enzymatic activity before and after RAC1 overexpression (mean ± SEM, n = 3, one-way ANOVA, $P > 0.05$). (F) Statistical analysis of *α*-SMA immunofluorescence expression in HRMCs (mean ± SEM, n = 3, one-way ANOVA, $^{*}P < 0.05$). (G, H) Illustrative images and statistical data depicting the THBS1 within HRMCs following TNF*α* stimulation (Scale bar=100 μm, mean ± SEM, n = 3, one-way ANOVA, $^{*}P < 0.05$, $^{**}P < 0.01$, $^{****}P < 0.0001$, ns no significance).

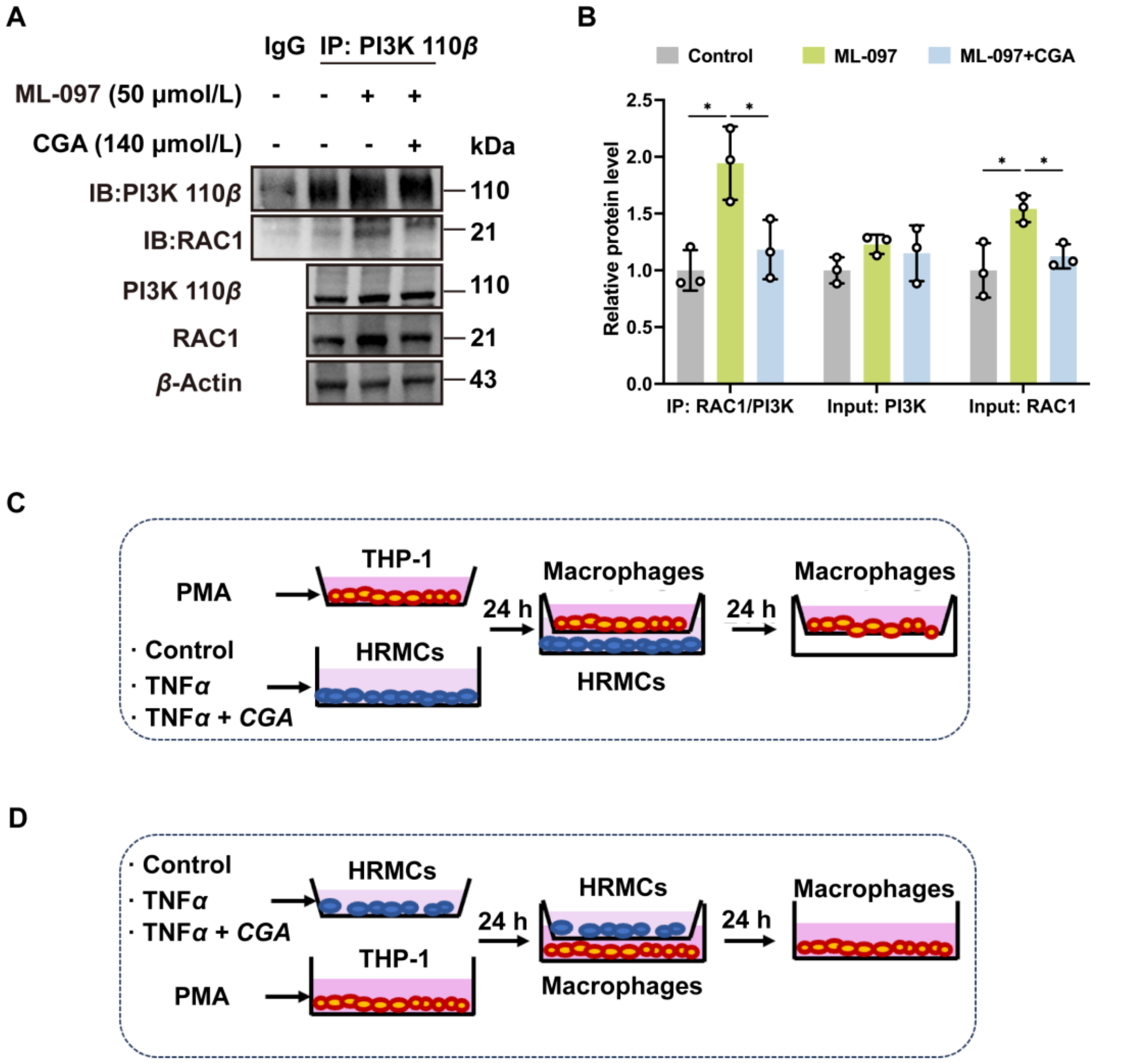

Figure S7 Co-IP assay for the binding ability between RAC1 and PI3K 110β, and schematic of the cell co-culture system. (A-B) Co-IP Assay for the Binding Ability Between RAC1 and PI3K 110β Under Treatment with the RAC1 Agonist ML097 (mean ± SEM, n = 3, one-way ANOVA, $^{*}P < 0.05$). (C) Schematic of the cell co-culture system for the migration assay. (D) Schematic of cell co-culture system for Co-IP and macrophage inflammatory factor release assay.

Table S1 Summary of primer sequences

Primers	Species	Forward 3'-5'	Reverse 5'-3'
PCNA	Homo	AACCTGCAGAGCATGGACTC	TATCCGCGTTATCTTCGGCC
KI67	Homo	ACAGACCTCAAGAGCTTGCC	CCAGGGATGCCTTCAACTGT
Il6	Rat	TGGAGTTCCGTTTCTACCTG	TAGGGTTTCAGTATTGCTCT
IL6	Homo	GTAGCCGCCCCACACAGA	CATGTCTCCTTTCTCAGGGCTG
Il1b	Rat	TGTGAAATAGCAGCTTTCGAC	CACACGTTTTCTTTGACCAG
IL1β	Homo	TTGACGGACCCCAAAAGAT	GAAGCTGGATGCTCTCATCTG
Tnfa	Rat	TTCCCAAATGGGCTCCCTCT	GTGGGCTACGGGCTTGTCAC
TNF*α*	Homo	TGGCCCAGACCCTCACACTCAG	ACCCATCGGCTGGCACCACT
Thbs1	Rat	TGGCATCATTTGCGGAGAG	TTTTCATTAGGCCAGCCGTC
GAPDH	Homo	TCAAGAAGGTGGTGAAGCAG	AGGTGGAAGAATGGGAGTTG
18S	All species	GTAACCCGTTGAACCCCATT	CCATCCAACGGTAGTAGCG

Table S2 Mass spectrometer search parameters

Item	Parameter
Database	Swissprot
Taxonomy	Homo sapiens
Enzyme	Trypsin
Fixed modifications	Carbamidomethyl (C)
Variable modifications Acetyl (Protein N-term), Oxidation (M)	Max Missed Cleavages 2
Max Missed Cleavages	2
Target FDR	0.01
Minimum Peptide Length	6
Peptide mass tolerance	10 ppm
MS/MS tolerance	±0.02 Da
